# Incorporation of a skeletal muscle-specific enhancer in the regulatory region of *Igf1* upregulates IGF1 expression and induces skeletal muscle hypertrophy

**DOI:** 10.1038/s41598-018-21122-5

**Published:** 2018-02-09

**Authors:** Yunlong Zou, Yanjun Dong, Qingyong Meng, Yaofeng Zhao, Ning Li

**Affiliations:** 1 State Key Laboratory for Agrobiotechnology, China Agricultural University, Beijing, 100193 P. R. China; 2 College of Veterinary Medicine, China Agricultural University, Beijing, 100193 P. R. China

## Abstract

In this study, we upregulated insulin-like growth factor-1 (IGF1) expression specifically in skeletal muscle by engineering an enhancer into its non-coding regions and verified the expected phenotype in a mouse model. To select an appropriate site for introducing a skeletal muscle-specific myosin light chain (MLC) enhancer, three candidate sites that exhibited the least evolutionary conservation were chosen and validated in C2C12 single-cell colonies harbouring the MLC enhancer at each site. IGF1 was dramatically upregulated in only the site 2 single-cell colony series, and it exhibited functional activity leading to the formation of extra myotubes. Therefore, we chose site 2 to generate a genetically modified (GM) mouse model with the MLC enhancer incorporated by CRISPR/Cas9 technology. The GM mice exhibited dramatically elevated IGF1 levels, which stimulated downstream pathways in skeletal muscle. Female GM mice exhibited more conspicuous muscle hypertrophy than male GM mice. The GM mice possessed similar circulating IGF1 levels and tibia length as their WT littermates; they also did not exhibit heart abnormalities. Our findings demonstrate that genetically modifying a non-coding region is a feasible method to upregulate gene expression and obtain animals with desirable traits.

## Introduction

Many genetically modified (GM) animals with desirable phenotypes have been generated for agricultural and biomedical applications. However, most traditional methods for creating transgenic animals usually involve the random insertion of the coding cassette into the genome^1–5^. Although this technique seems straightforward, the copy number and insertion locus cannot be accurately controlled, leading to unstable gene expression^6,7^. Site-specific incorporation of the expressing construct is an improved option, but this approach is very arduous, particularly when the genomic DNA sequences of genes that span large genomic regions are used. Although cDNA can be used as a substitute, it may lack the complex non-coding regulatory elements that remain undefined but substantially contribute to expression, rendering cDNAs unfavourable for high expression levels in most cases^8–10^.

Naturally occurring mutations in the regulatory regions of genes can have large effects on phenotypic variation^11–14^. For example, a single-nucleotide polymorphism substitution in intron 3 of the insulin-like growth factor (*IGF*)-2 gene in pigs causes a three-fold increase in its mRNA levels in skeletal muscle, generating a 3–4% increase in meat production^12,13^. Moreover, a G to A transition in the 3′ untranslated region of the myostatin gene in Texel sheep creates a target site for mir1 and mir206, inhibiting translation of the myostatin gene and subsequently contributing to muscular hypertrophy^14^. However, most studies focus on naturally occurring mutations, and reports of artificially editing non-coding genomic sequences to improve production traits in live animals are rare.

As key components of non-coding regions, enhancers stimulate gene expression in a spatiotemporal-specific manner independent of the orientation or distance to the promoter. Moreover, enhancers are active in heterologous sequence contexts^15,16^. Thus, we speculated that the site-specific insertion of a tissue-specific enhancer might upregulate target gene expression physiologically and spatiotemporally, consequently preserving its original regulatory network and decreasing the abnormalities induced by the forced transgene expression.

IGF1 is a key factor regulating skeletal muscle development and growth, and the muscle-specific upregulation of IGF1 leads to muscle hypertrophy^17–21^. Until recently, *Igf1* transgenic mice have not been generated using the entire genomic DNA sequence, which is >70 kb. To our knowledge, the existing *Igf1* transgenic mouse models have all been created by randomly inserting a cDNA construct encoding either different isoforms of the pre-pro-peptide^21–23^ or the mature form^24–26^.

Here, we used the *Igf1* gene as an example in this proof-of-concept report. We asked whether *Igf1* expression was upregulated by including an enhancer in the non-coding regions upstream of its transcriptional start site. In addition, we investigated whether this genetic modification generates the expected phenotype in the mouse model.

## Results

### A screen for an efficient candidate enhancer by luciferase reporter assays

The MLC (myosin light chain)^27–30^ and MCK (muscle creatine kinase)^31–34^ enhancers are extensively characterized muscle-specific enhancers; therefore, we chose them as candidates for insertion into the upstream regions of the *Igf1* transcriptional start site. The two enhancers exert their functions in differentiated myotubes rather than undifferentiated myoblasts^28,33^. To evaluate the effects of the MLC and MCK enhancers on the *Igf1* promoter, we performed luciferase reporter assays.

**Figure 1:**
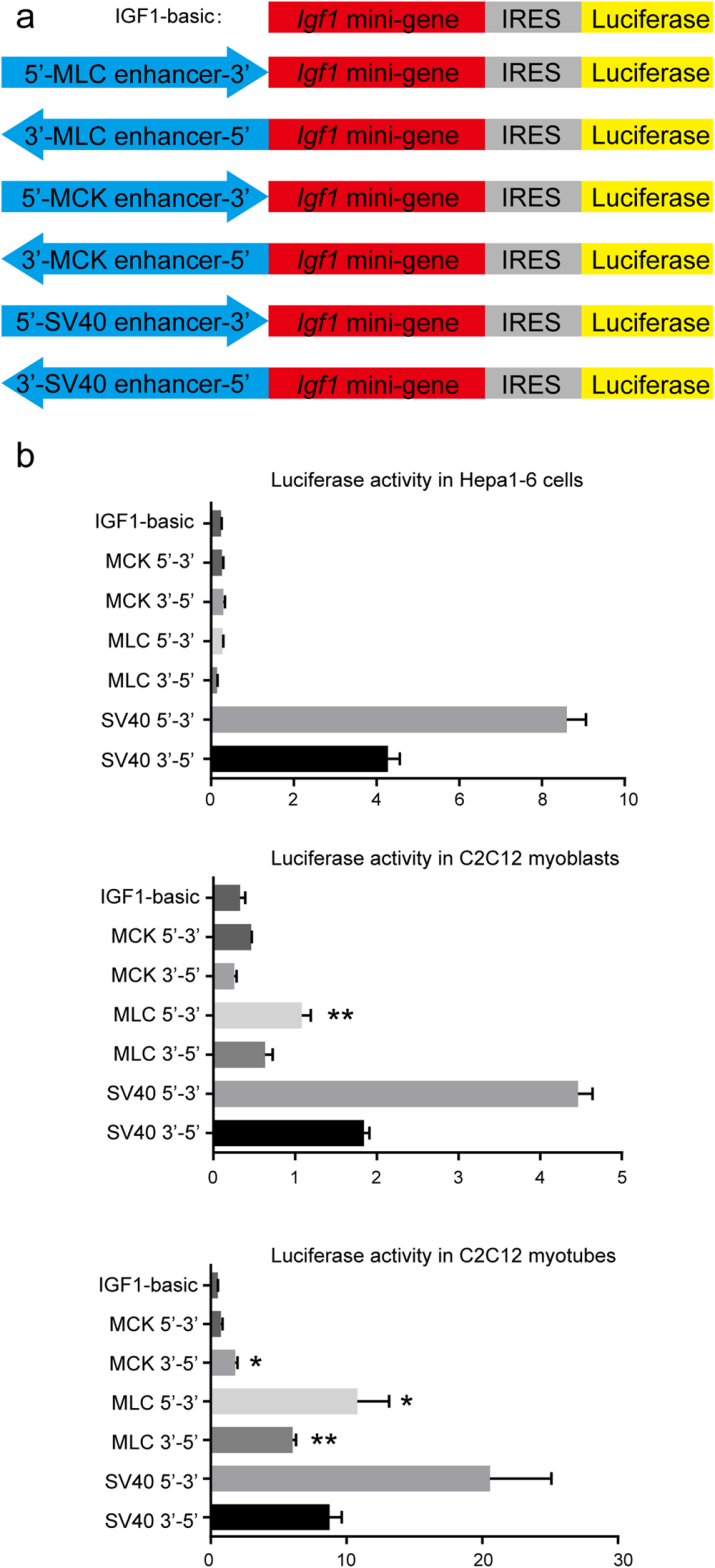
Confirmation of the activity of candidate enhancers by luciferase assays. **(a**) A schematic of luciferase reporter vectors. The enhancer sequences are linked to the *Igf1* mini-gene in both orientations. The yellow, grey, and red blocks represent the luciferase coding cassette, the IRES element, and the *Igf1* mini-gene cassette, respectively. The blue arrows represent the enhancer sequences and indicate the orientation with respect to the *Igf1* mini-gene. **(b)** The luciferase expression levels of the indicated constructs in Hepa1–6 cells (top), C2C12 myoblasts (middle), and C2C12 myotubes (bottom). The results are representative of three independent experiments. Bars represent the mean values, and the error bars represent the standard error of the mean (SEM). *P < 0.05 between indicated reporter vectors and the IGF1-basic construct; **P < 0.01 between indicated reporter vectors and the IGF1-basic construct. Statistical significances were analysed by Student’s t-test.

For luciferase reporter vector construction, we used the *Igf1* mini-gene, which contains exon 1, intron 1, exon 2, intron 2, and part of exon 3, as the promoter. In addition, an IRES element was used to connect the luciferase gene and the *Igf1* mini-gene, which gave rise to several polypeptides (Fig. 1a).

The luciferase expression of the IGF1-basic construct was regarded as background level (Fig. 1a), and the SV40 enhancer was chosen as a positive control (Fig. 1a). In the differentiated C2C12 myotubes, the MLC enhancer dramatically increased luciferase expression by 12 to 20-fold above that of the background level in both orientations (Fig. 1b). The activity of the MLC enhancer in the 5′-3′ orientation was low but significant in C2C12 myoblasts (Fig. 1b), a result that may be explained by a small quantity of differentiated myotubes. The MLC enhancer was essentially inactive in Hepa1–6 cell lines (Fig. 1b). Compared with the background expression level, the MCK enhancer showed a small but significant increase in luciferase expression in differentiated C2C12 myotubes but no difference in either C2C12 myoblasts or the Hepa1–6 cell line (Fig. 1b). Hence, we selected the MLC enhancer for subsequent experiments.

### A screen for the candidate insertion site and further validation in C2C12 single-cell colonies

To search for an appropriate integration site for the MLC enhancer, we divided the region 100 kb upstream the *Igf1* transcriptional start site into non-overlapping 50 bp windows, and calculated average PhastCons score from the UCSC database for each window^35^. We chose the three candidate sites with the lowest PhastCons scores, or say the least evolutionarily conserved sites, to avoid functional *cis*-regulatory elements that are generally evolutionarily conserved^36^.

**Figure 2:**
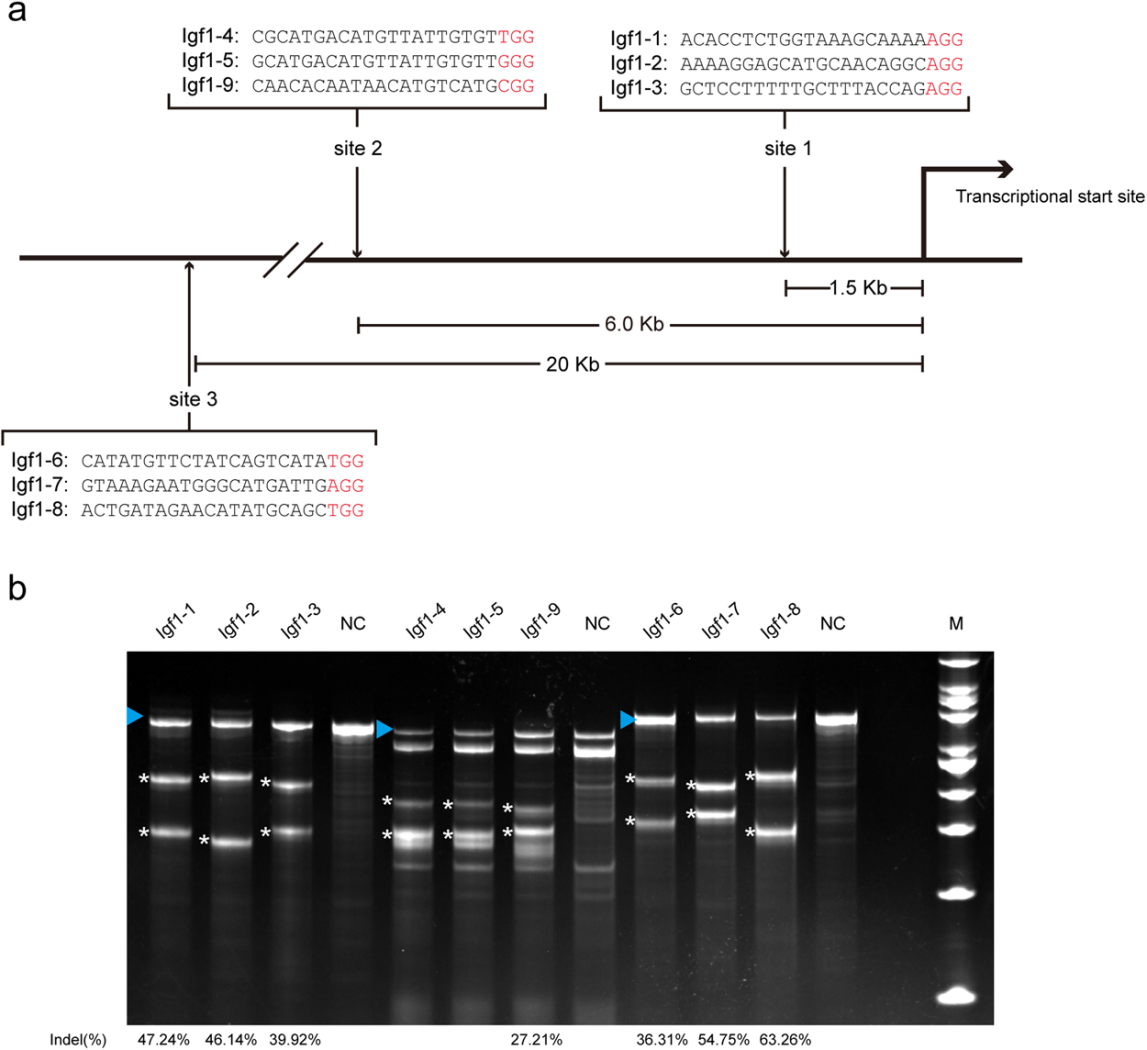
Evaluation of sgRNAs targeting the three candidate insertion sites. **(a)** Schematic of the sgRNAs targeting the three candidate insertion sites. The protospacer-adjacent motifs (PAM) are labelled in red. The distance between each candidate insertion site and the transcriptional start site is shown. **(b)** T7EN1 assay detection of the efficiency of Cas9-mediated cleavage at the indicated targets. The blue triangles indicate the locations of the PCR products before T7EN1 digestion. The asterisks indicate cleaved PCR amplicon fragments of the expected sizes after T7EN1 digestion. The extra blots in lanes of the sgRNAs targeting the site 2 have no relation to the gene editing events and may arise from the SNPs existing in the C2C12 genome. The sgRNAs Igf1–1, Igf1–9 and Igf1–7 were used to generate double-stranded breaks to stimulate homologous recombination in each of the three respective candidate sites. NC: WT C2C12.

**Figure 3:**
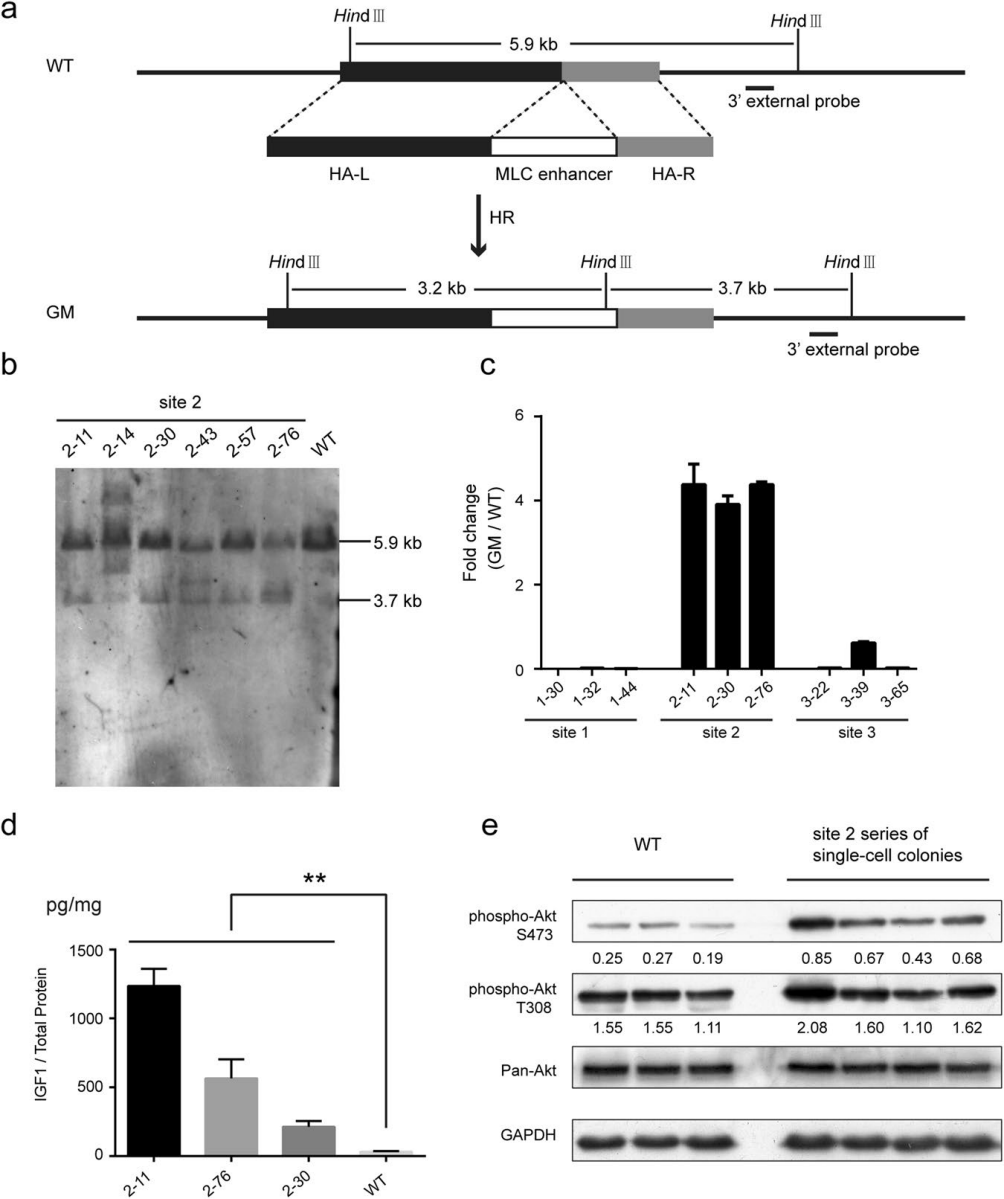
Evaluation of the ability of the candidate sites to support IGF1 expression in C2C12 single-cell colonies. **(a)** Schematic of the strategy used to knock in the MLC enhancer by homologous recombination (HR). The site 2 is chosen as an example; the strategy was the same for the site 1 and site 3. The black and grey blocks represent the 5′ long homology arm (HA-L) and 3′ short homology arms (HA-R), respectively. The white blocks represent the incorporated MLC enhancer. The restriction enzyme used for Southern blot analysis is shown. The 3′ external probe is shown as a short black line. **(b)** Southern blot analysis of the incorporated MLC enhancer in the site 2. Genomic DNA of the site 2 series of single-cell colonies and the WT control was digested with *Hin*dIII and then hybridized with the 3′ external probe. The expected fragment sizes are: WT, 5.9 kb; GM, 3.7 kb. **(c)** Real-time quantitative PCR analysis of the relative expression levels of *Igf1* mRNA in myotubes of the site 1, site 2, site 3 series of single-cell colonies. The results were displayed as fold changes relative to the expression levels in WT C2C12 myotubes using the 2^−△△CT^ method. Values are mean ± SEM of three independent experiments. **(d)** ELISA analysis of the IGF1 protein levels in myotubes of the site 2 series of single-cell colonies and the WT control. The total IGF1 levels are normalized to the total protein levels. Values are mean ± SEM of three independent experiments. **(e**) Western blot analysis of the Akt phosphorylation levels in myotubes of the site 2 series of single-cell-colonies and the WT control. Antibodies against phosphorylated Akt (Ser473), Akt (Thr308), and total Akt were used. GAPDH was used as a loading control. The ratio of the phosphorylated Akt (Ser473)/Pan-Akt and the phosphorylated Akt (Thr308)/Pan-Akt were determined by calculating the blot intensities and are shown below each respective blot. All gels/blots were run under the same experimental conditions. Shown are cropped gels/blots (Full-length gels/blots with indicated cropping lines are shown in Supplementary Figure S10).

To evaluate the candidate sites for incorporation and to further investigate the MLC enhancer in the C2C12 cell line, we designed nine sgRNAs targeting the three candidate sites (three for each), which are located at distances of 1.5 kb, 6 kb, and 20 kb from the *Igf1* transcriptional start site, respectively (Fig. 2a). A high cleavage efficiency of the sgRNAs targeting the three candidate sites was detected in C2C12 cells by T7EN1 assays (Fig. 2b). Then, successful homologous recombination in each of the three candidate sites was confirmed in a pool of cells co-transfected with the donor vector, a plasmid expressing the Cas9 protein and the sgRNAs targeting each respective site, and a green fluorescent protein (GFP)-expressing vector, which was used to enrich GFP^+^ cells by flow cytometry (Fig. 3a and Supplementary Fig. S1).

To obtain C2C12 single-cell colonies that incorporated the MLC enhancer for further investigation, we co-transfected the donor vector, a plasmid expressing the Cas9 protein and the sgRNAs targeting each respective site. Then, single-cell colonies were generated through a limiting dilution technique and examined for 5′ and 3′ integration by PCR. For the site 1, 4 out of 55 C2C12 single-cell colonies showed correct integration, whereas no positive C2C12 single-cell colonies were obtained for the site 2 and site 3. Therefore, to enrich for targeted cells, a GFP-coding cassette was added to the original Cas9 and sgRNA expressing vectors for flow cytometry sorting of GFP^+^ cells (Supplementary Fig. S2). The results showed that 11 out of 96 single-cell colonies tested for site 2 and 5 out of 48 single-cell colonies tested for site 3 were successfully targeted. The insertion of the MLC enhancer into site 2 was further confirmed by Southern blot analysis (Fig. 3a and b). Then, we examined the off-target effects in four single-cell colonies of site 2, and no mutations were detected in the ten most likely off-target sites by Sanger sequencing (Supplementary Fig. S3).

**Figure 4:**
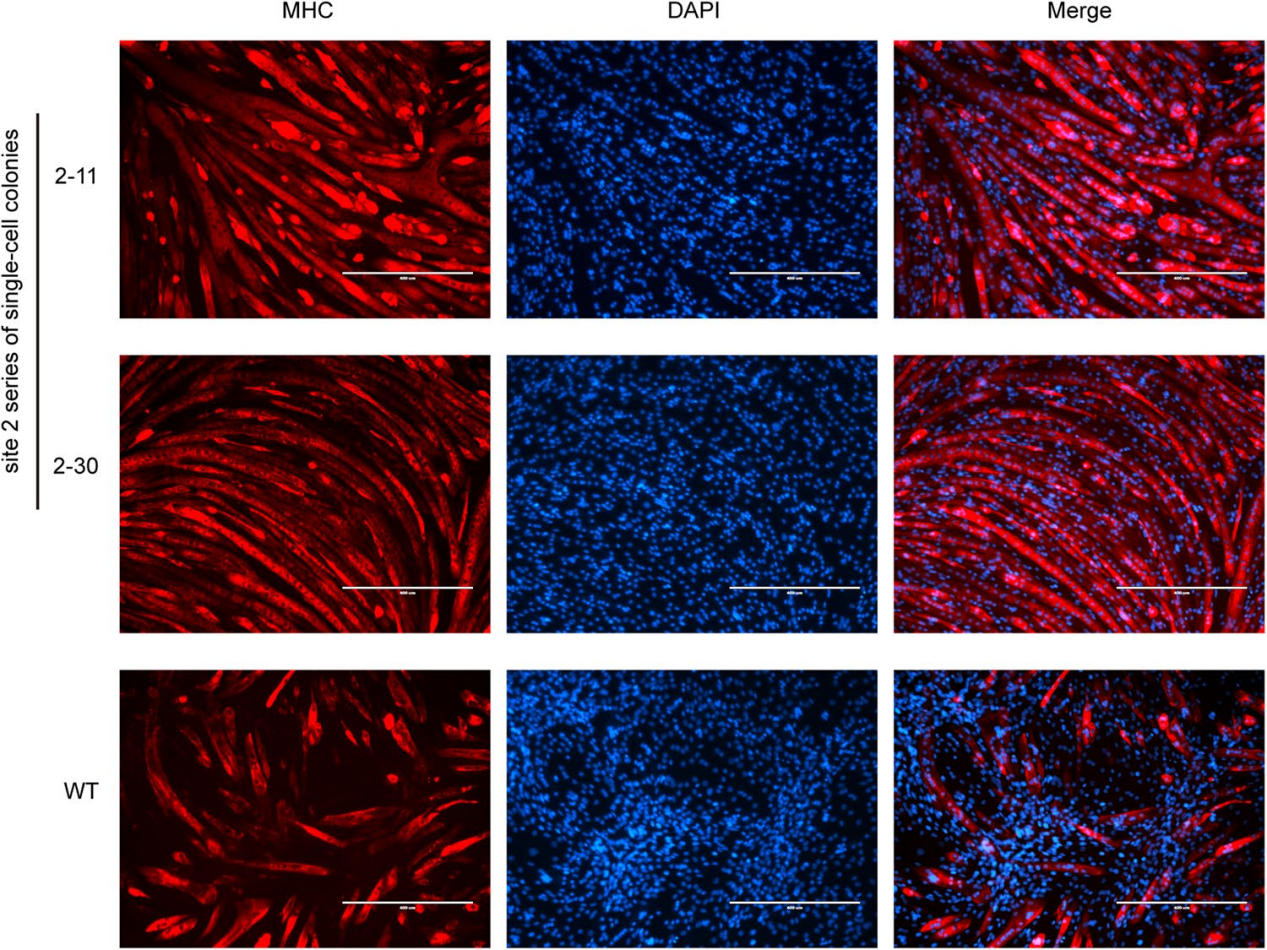
Detection of the muscle hypertrophy phenotype in the site 2 series of single-cell colonies. Immunofluorescence staining of MHC in myotubes of the site 2 series of single-cell colonies and the C2C12 WT control.

To test the ability of each of the three candidate sites to support IGF1 expression, we evaluated the *Igf1* mRNA expression levels in the three series of single-cell colonies. Compared with the WT control, only the site 2 series of single-cell colonies exhibited a 4-fold higher *Igf1* expression level, whereas the *Igf1* expression level in both the site 1 and site 3 series was downregulated (Fig. 3c), thus suggesting a disruption of important *cis*-regulatory elements. A significant increase in IGF1 protein levels was also detected in three separate single-cell colonies of the site 2 series compared with the levels in the WT C2C12 controls (Fig. 3d). To determine whether the elevated IGF1 amplified the IGF1/Akt pathway, which is closely related to muscle hypertrophy, we examined the phosphorylation levels of Akt. Both the phosphorylation levels of Akt (Ser473) and Akt (Thr308) were higher in most of the site 2 series of single-cell colonies compared with the WT C2C12 cells (Fig. 3e). Moreover, the site 2 series of single-cell colonies more readily differentiated into myotubes **(**Supplementary Fig. S5**)**, and more myotubes were detected by immunofluorescence staining of myosin heavy chain (MHC) (Fig. 4).

Together, these results showed that site 2 was an ideal site for incorporation of the MLC enhancer to drive *Igf1* expression in C2C12 cells.

### Generation of GM mice with the MLC enhancer incorporated in site 2

**Figure 5:**
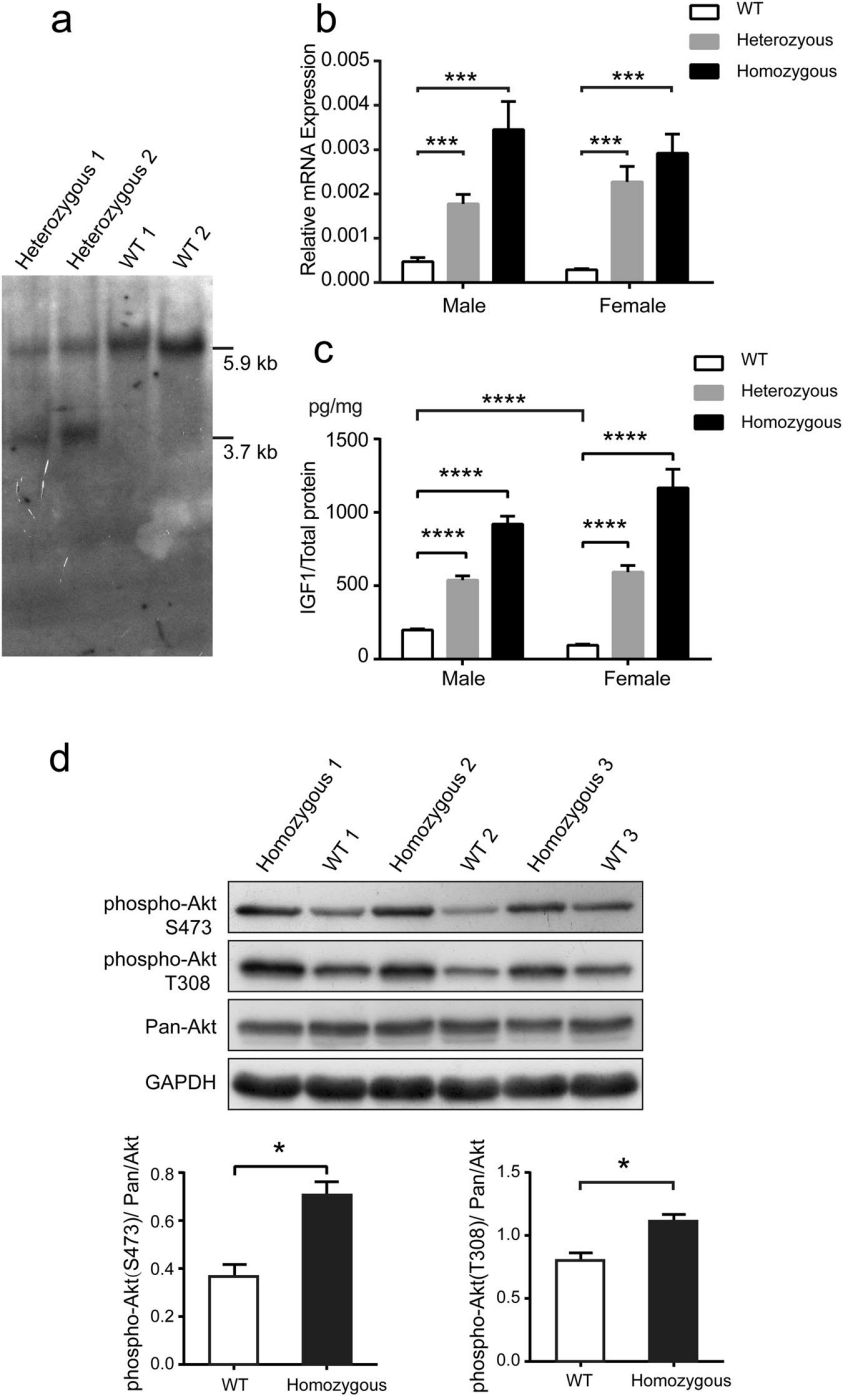
Analysis of IGF1 expression levels in the genetically modified mouse model. **(a)** The strategy for Southern blot analysis of incorporation of the MLC enhancer in GM mice is the same as that shown in Fig. 2c. Genomic DNA of heterozygous GM mice and WT control was digested with *Hin*dIII and then hybridized with the 3′ external probe. The expected fragment sizes are: WT, 5.9 kb; GM, 3.7 kb. **(b)** Real-time PCR analysis of *Igf1* mRNA levels in the gastrocnemius muscle of two-month-old GM mice and WT littermates. (n = 11–13 per group). **(c)** ELISA analysis of total IGF1 protein levels in the gastrocnemius muscle of two-month-old GM mice and WT littermates. The total IGF1 protein levels were normalized to the total protein levels. (n = 10–13 per group). **(d)** Western blot analysis of the Akt phosphorylation levels in the gastrocnemius muscle of one-month-old female GM mice and WT littermates. Antibodies against phosphorylated Akt (Ser473), Akt (Thr308), and total Akt were used. GAPDH was used as a loading control. The ratio of phosphorylated Akt (Ser473)/Pan-Akt and phosphorylated Akt (Thr308)/Pan-Akt were determined by calculating the intensities of the blots and are shown below. All gels/blots were run under the same experimental conditions. Shown are cropped gels/blots (Full-length gels/blots with indicated cropping lines are shown in Supplementary Figure S10). Bars depict mean values, and error bars represent SEM. *P < 0.05, **P < 0.01, ***P < 0.0001, ****P < 0.000001. Statistical significances were analysed by Student’s t-test.

To extend our study from cell lines to a whole animal context, we generated genetically modified mice by injecting Cas9 mRNA, sgRNA targeting site 2 and the circular donor plasmid into mouse zygotes (Table 1). The successful incorporation of the MLC enhancer was confirmed in F0 mice by PCR across the 3′ and 5′ junctions **(**Table 1**)** and was confirmed in their offspring by Southern blot analysis (Figs 3a and 5a). The efficiency of homologous recombination was 37.50% (Table 1). The integration observed in the eight positive founders that were chosen for offspring production were transmitted through the germline to the F1 progeny.

**Table 1:** Generation of a genetically modified mouse model.

Experiment times	Number of Injected zygotes	Number of recipients	Number of pregnant recipients	Number of newborns	Pregnancy rate	Live-birth rate	Number of the positives	F0 positive Litters	Positive rate
1	250	10	4	13 (5♂ + 8♀)	40.00%	5.20%	3 (1♂ + 2♀)	**1#**♂, 6#♀, 10#♀	23.07%
2	160	8	3	19 (11♂ + 8♀)	37.50%	11.88%	9 (5♂ + 4♀)	**17#**♂, **18#**♂, **19#**♂, 21#♂, **22#**♀, **26#**♀, 27#♀, 29#♂, **31#**♀	47.37%
Total	410	18	7	32 (16♂ + 16♀)	38.89%	7.80%	12 (6♂ + 6♀)		37.50%

5 ng/µl Cas9 mRNA, 2.5 ng/µl sgRNAs and 5 ng/µl donor vector targeting the site 2 locus were co-injected into zygotes of the C57BL/6 mouse. To circumvent random integrations, a circular donor vector was used instead of a linear one. The sex distributions of the total number of newborns and positive newborns are shown in parentheses respectively. The F0 positive mice used to produce offspring are labelled in bold.

### Upregulation of *Igf1* expression and the stimulation of downstream signal pathway in skeletal muscle

To investigate whether the incorporation of the MLC enhancer increased *Igf1* transcription and thus increased protein accumulation *in vivo*, *Igf1* mRNA and protein expression levels were examined in two-month-old mice. In the gastrocnemius (GA) muscle, the *Igf1* mRNA levels in both male and female GM mice showed a significant increase relative to the levels in their WT littermates (Fig. 5b). Two-way ANOVA analysis demonstrated that the genotype factor significantly affected the results (P < 0.0001), while the sex factor did not (P = 0.7959), and the interaction of the two factors also showed no significant effects (P = 0.3234). Then, we further examined the IGF1 protein levels, which also sharply increased in both male and female GM mice compared with their sex-matched WT littermates (Fig. 5c). Interestingly, the IGF1 protein levels increased by a greater extent in the female GM mice than the male GM mice, as compared with their sex-matched WT littermates (Fig. 5c). However, the male WT mice demonstrated greater endogenous IGF1 protein levels compared with those of the female WT mice (P < 0.00000001) (Fig. 5c). Two-way ANOVA analysis demonstrated that the genotype factor significantly affected the results (P < 0.0001), but the sex factor had no significant effects on the results (P = 0.1743). However, the interaction of the two factors showed significant effects on the results (P < 0.05).

Significantly increased levels of the phosphorylation of Akt (Ser473) and Akt (Thr308) were detected at one month in the GA muscles of female GM mice (Fig. 5d), but not at two months (Supplementary Fig. S4). This result was consistent with a previous report showing that a growth stimulus is required for the Akt activation in respond to elevated IGF1^23^. Besides, a higher phosphorylation level of Akt (Ser473) in the GA muscle of 1-month-old male GM mice was also detected (Supplementary Fig. S6**)**.

Furthermore, the results demonstrated that in the GA muscle of both male and female GM mice, the phosphorylation levels of mTOR (Ser2448), P70S6K (Thr389), and S6 (Ser240/244) were increased, and the phosphorylation levels of 4E-BP1 (Ser65) were decreased. Thus, the IGF1/Akt pathway was stimulated (Supplementary Fig. S6**)**.

### Elevated IGF1 leads to skeletal muscle hypertrophy in female GM mice

**Figure 6:**
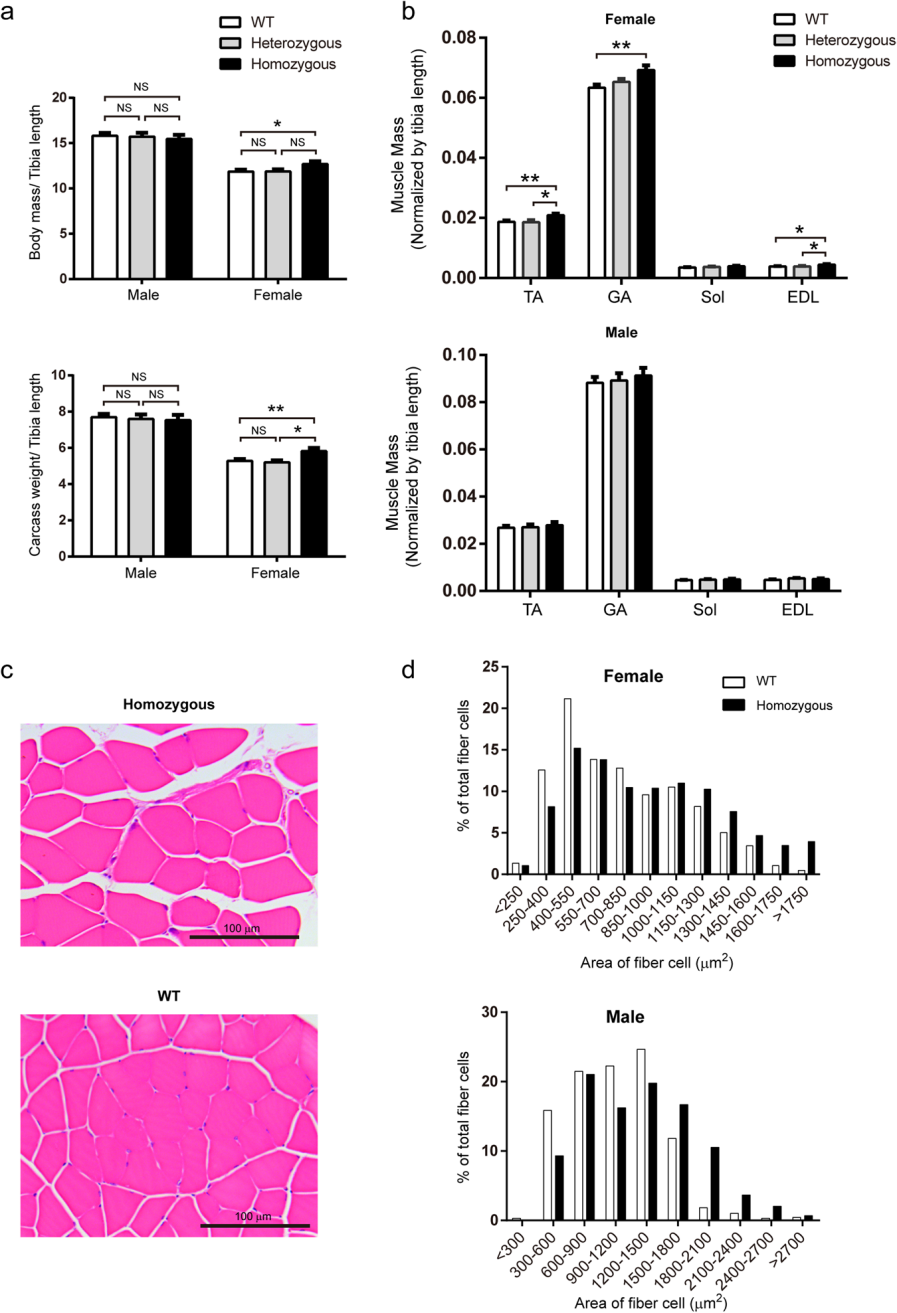
Detection of skeletal muscle hypertrophy in a genetically modified mouse model. **(a)** Average body mass (top) and carcass weight (bottom) of the three-month-old GM mice and sex-matched WT littermates (n = 8–15 per group). The body mass and carcass weight were normalized to the length of the tibia, respectively. **(b)** Determination of the muscle weight of the three-month-old GM mice and WT littermates (top, female mice; bottom, male mice). The data indicate the average weight of the muscles from the left and right sides. The muscle weight is normalized to the length of the tibia. (n = 10 for female homozygous GM mice, n = 8 for female heterozygous GM mice, and n = 15 for female WT mice; n = 10 for male homozygous GM mice, n = 8 for male heterozygous GM mice, and n = 12 for male WT mice). **(c)** Representative images of H&E-stained cross-sections of the TA muscle from three-month-old female GM mice and WT littermates. **(d)** CSA frequency distribution of myofibres in the TA muscle from three-month-old GM mice and sex-matched WT littermates (sections from at least five mice per group). Bars depict mean values, and error bars represent SEM. *P < 0.05, **P < 0.01. Statistical significances were analysed by Student’s t-test.

At the age of three months, the homozygous female GM mice demonstrated a significantly heavier body weight and carcass weight compared with that of sex-matched WT mice; in contrast, no differences were detected for the male mice (Fig. 6a).

To determine whether the increased body weight resulted from increased muscle mass, we dissected out the GA, soleus (Sol), extensor digitorum longus (EDL), and tibialis anterior (TA) muscles from the male and female mice at three months of age. The homozygous female GM mice showed a significant increase in TA (11.86%), GA (9.23%), Sol (11.68%) and EDL (16.75%) muscle weights compared with those of the sex-matched WT littermates (Fig. 6b). In addition, compared with the WT littermates, a similarly increasing trend was observed in the weights of the TA (4.18%), GA (3.55%), Sol (5.74%) and EDL (8.02%) muscles in homozygous male GM mice, though these results were not statistically significant (Fig. 6b). One possible explanation for the less conspicuous phenotype of male GM mice may be that the smaller increase fold of IGF1 protein in male GM mice than that in the female GM mice compared with sex-matched WT littermates (Fig. 5c).

To determine whether increased muscle mass was due to the hyperplasia and/or hypertrophy of myofibres, we examined the total number of myofibres in the tibialis anterior (TA) muscle but detected no differences between GM mice and their WT littermates (Supplementary Figure S7). Then, we measured the cross-sectional area (CSA) of the TA and GA muscles in three-month-old GM mice and their WT littermates. Compared with sex-matched WT littermates, the average CSA of the TA muscle increased by 18.6% and 17.8% in male and female GM mice, respectively (Fig. 6c). An increase in the CSA of the GA muscle in GM mice was also observed. The CSA distribution for the TA muscle demonstrated that the relative proportion of larger myofibres in the GM mice was greater, and very large myofibres, which were not ordinarily found in WT littermates, were also detected (Fig. 6d).

To examine whether elevated IGF1 levels exhibited fibre-type-specific effects on muscle hypertrophy, we performed immunofluorescence by staining for MHCI, MHCIIa and MHCIIb and measured the CSA of each fibre type. We found that the CSA of MHC type IIb fibres exhibited a significant increase in the muscles of GM mice; however the CSA of MHC I and MHC IIa type fibres exhibited only a minor increase (Supplementary Figure S8**)**. To examine whether fibre-type-specific hypertrophy was driven by fibre-type-specific IGF1 pathway stimulation, we dissected the EDL muscle, a typical fast-twitch muscle, and the Sol muscle, a slow-twitch muscle, to examine S6 phosphorylation (Ser240/244) in each muscle individually. Higher levels of S6 phosphorylation (Ser240/244) were detected in the EDL muscle of GM mice, whereas no significant differences were detected in the Sol muscle of the mice (Supplementary Figure S9**)**. The increased S6 activation (Ser240/244) in the EDL muscle of GM mice might have resulted from higher *Igf1* expression in fast-twitch fibres, because the MLC enhancer is active predominately in fast IIb fibres^21^.

### Assessment of the systemic effect of elevated IGF1 in skeletal muscle

To investigate whether the increased levels of IGF1 in skeletal muscle had a systemic effect, we detected the circulating IGF1 levels and examined the heart and bone, which are closely related to skeletal muscle.

**Figure 7:**
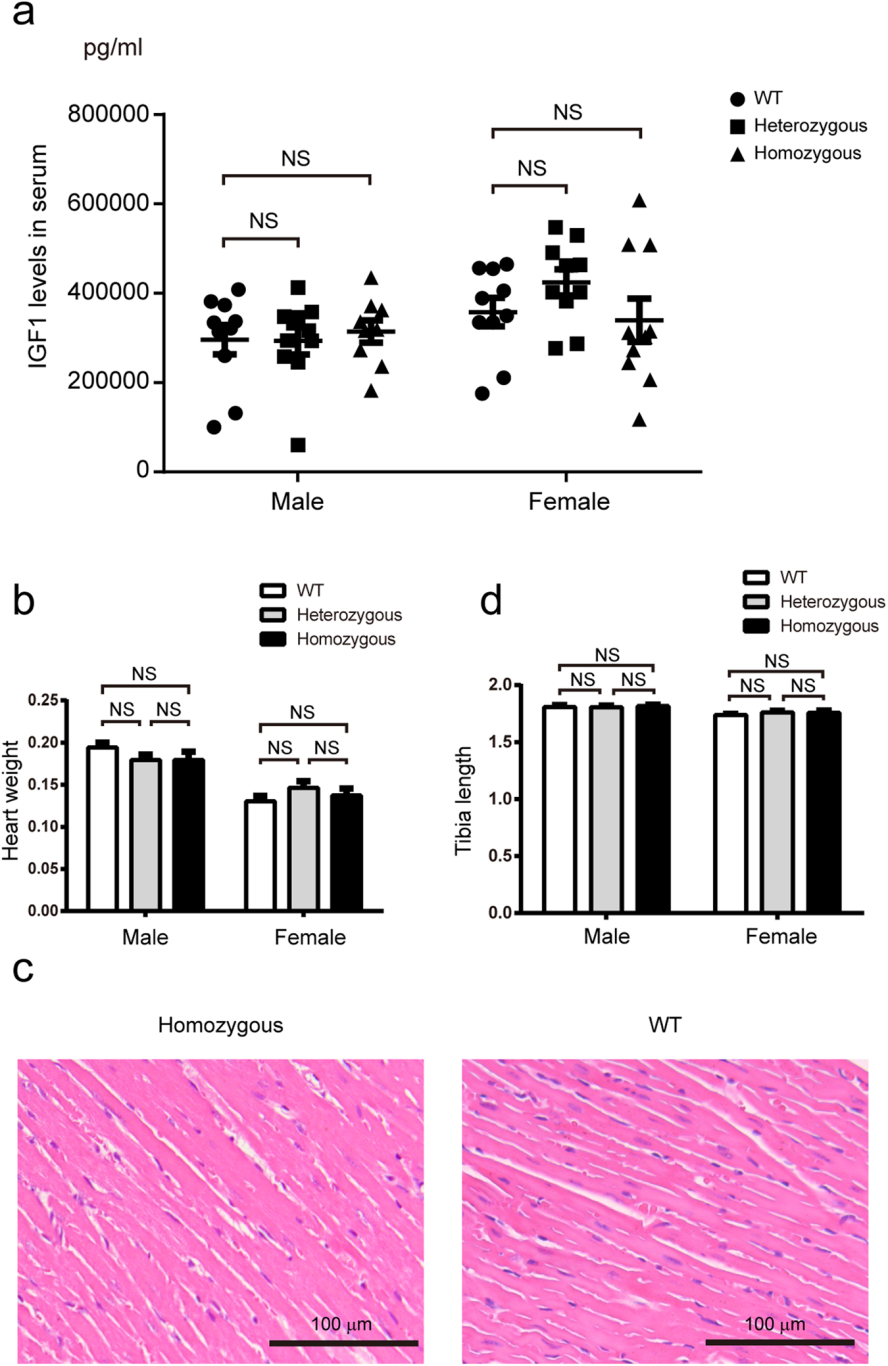
Evaluation of the systemic effect of elevated IGF1 in skeletal muscle. **(a)** ELISA analysis of the total IGF1 levels in serum from two-month-old GM mice and WT littermates (n = 9–10 per group). **(b)** Determination of the weight of the heart from three-month-old GM mice and WT littermates (n = 7–13 per group). **(c)** No pathological changes were detected by H&E analysis of the longitudinal section of hearts from three-month old GM mice and WT littermates. **(d)** Comparison of the tibia lengths of three-month-old GM mice and WT littermates. (n = 8–15 per group). Bars depict mean values, and error bars represent SEM. NS, not significant. Statistical significances were analysed by Student’s t-test.

There were no differences in the circulating levels of IGF1 between the GM mice and their sex-matched WT littermates (Fig. 7a). In the GM mice, no differences in the weight and size of the heart were detected, and the hearts showed no pathological changes (Fig. 7b and c). The tibia lengths were measured, and no differences were detected in either male or female GM mice compared with their sex-matched WT littermates (Fig. 7d).

Together, these results showed that the increase in IGF1 expression was restricted to skeletal muscle, and the effect of the increased IGF1 in the skeletal muscle did not cause systemic perturbations in the GM mice.

## Discussion

In our study, we established a method for altering gene expression that affects traits via the genomic editing of a non-coding sequence. By incorporating the MLC enhancer into the site 2 upstream of the *Igf1* promoter, IGF1 was successfully upregulated in both the C2C12 cell line and in the skeletal muscle of GM mice. The GM mice developed normally and exhibited skeletal muscle hypertrophy with no systemic perturbations.

In our mouse model, we circumvented the disadvantages of the lack of important *cis*-regulatory elements not included in cDNA sequences and addressed the difficulties of site-specifically incorporating a large genomic sequence (>70 kb) when generating transgenic animals. By exploiting the endogenous regulatory mechanisms of *Igf1*, the addition of a skeletal muscle-specific enhancer simply and elegantly increased expression in a physiological and spatiotemporal manner.

In our mouse model, the IGF1 expression was not as high as in previous reports. Two possible explanations for this outcome are (1) different genetic mouse backgrounds might affect the phenotype differently^37,38^; and (2) previous studies have used multiple copies of the expression cassette, whereas in our study, only a single copy of the MLC enhancer was used. To increase IGF1 upregulation, combinations of natural enhancers or powerful, synthetic artificial enhancers can be used. Furthermore, the gene expression pattern can be accurately and predictably adjusted by substituting enhancers with different tissue specificity and potency; this strategy is both versatile and flexible.

Both male and female GM mice exhibited significant upregulation of *Igf1* mRNA and IGF1 protein in skeletal muscle, demonstrating that we achieved the goal of our research. However, the *Igf1* mRNA and the IGF1 protein showed a greater increase in female GM mice than male GM mice compared with their sex-matched WT littermates. Moreover, female GM mice exhibited clear muscle hypertrophy, and although male GM mice exhibited a similar trend toward increased muscle weight and muscle hypertrophy, the differences were not statistically significant.

Two non-exclusive explanations might account for the differences between male and female GM mice. First, it has been reported that testosterone stimulates IGF1 expression in skeletal muscle^39–41^, and a previous report identified two androgen response elements within the human IGF1 upstream promoter that in *cis* activate IGF1 expression^42^. Using TF-Scan to screen the ∼800 bp of sequence flanking site 2, we did not detect any defined androgen response elements. Thus, we inferred that some uncharacterized skeletal muscle-specific androgen response elements potentiating the expression of *Igf1* exist in site 2 but may have been disrupted by insertion of the MLC enhancer. However, the positive effect of including the MLC enhancer might have predominated, compensating for the loss of potential androgen response element activity and leading to compromised upregulation of *Igf1*. The above speculation is supported by the fact that the *Igf1* mRNA levels in homozygous female GM mice were roughly 10-fold higher than sex-matched WT littermates, whereas the *Igf1* mRNA levels in homozygous male GM mice were about 7-fold higher. Second, in our study, we found that IGF1 protein levels were significantly higher in the muscles of WT male mice than WT female mice (P < 0.00000001) and inferred that the IGF1 signalling pathway may already be maximally stimulated in the intact male WT mice, making them resistant to further IGF1 upregulation and insensitive to the growth-promoting effects of IGF1.

Although the male GM mice did not exhibit conspicuous muscle hypertrophy, the pathway downstream of IGF1 was highly stimulated, similar to female GM littermates. We inferred that the lower expression of IGF1 protein would exhibit a delay exerting its function in male GM muscle, which might be less sensitive to IGF1 compared with female GM mice. In our subsequent research, we will re-examine the phenotype of male GM mice at a later time point.

With additional investigation into non-coding sequences and the development of genomic editing tools, the ability to create precise genetic modifications in animals to generate desired traits is becoming routine. As a proof-of-concept study in a mouse model, our strategy might provide insights into the production of livestock for agricultural and biomedical purposes.

## Methods

### Ethics Statement

All animal experiments in the present study were approved by the Animal Care and Use Committee of China Agricultural University (approval number: SKLAB-2012–01–08). All experimental procedures were performed in accordance with the guidelines for the Care and Use of Laboratory Animals of China Agricultural University.

### Construction of the Luciferase Reporter Vectors

We cloned the *Igf1* mini-gene containing exon 1, intron 1, exon 2, intron 2, and part of exon 3, 920 bp of the MLC enhancer^28^, 206 bp of the 5′ MCK enhancer^32^ and the SV40 enhancer by PCR. The results were confirmed by sequencing. We linked the *Igf1* mini-gene sequence to the luciferase gene in the PGL3 basic plasmid via an IRES element; the vector was named IGF1-basic and was used as the parent vector for the construction of other vectors. Starting with the IGF1-basic vector, we linked the MLC enhancer, the MCK enhancer, and the SV40 enhancer in either the 5′-3′ or 3′-5′ orientation.

### Cell culture

C2C12 and Hepa1–6 cells were maintained in growth medium consisting of Dulbecco’s Modified Eagle’s Medium (DMEM, Gibco), 10% fetal bovine serum (FBS, Gibco) and 1% penicillin-streptomycin. At a confluence of 60–70%, differentiation of the C2C12 cells was induced by replacement of the growth medium with differentiation medium (DMEM supplemented with 2% horse serum).

### T7EN1 assay

Three days after transfection, total DNA was extracted from C2C12 cells with a DNeasy Blood & Tissue Kit (Qiagen, Germany). The fragments flanking the Cas9 cutting sites were then PCR amplified. The primers used were shown in Supplementary Table S1. After annealing, the PCR products were digested with T7EN1 (NEB, cat. M0302S) at 37 °C for 1 h. PAGE electrophoresis was performed for fragment separation.

### Construction of the donor plasmids for homologous recombination

Through Gibson assembly (NEB, cat. E2611S), we constructed three targeting donor vectors, which contained 920 bp of the MLC enhancer flanked by ~2–3 kb of the 5′ and ~1 kb of the 3′ homology arms adjacent to each of the three candidate loci.

### Detection of homologous recombination in C2C12 single-cell colonies and mice by PCR

The primers used to detect positive C2C12 single-cell colonies were shown in Supplementary Table S2; to detect positive mice, the primers for amplifying across the 3′ junction of site 2 locus, across the 5′ junction of site 2 locus and across the MLC enhancer, were used.

### Transfection and luciferase assay

Transient transfections were performed with FuGENE® HD Transfection Reagent (Promega, USA) according to the manufacturer’s instructions. The Firefly luciferase plasmids were co-transfected with The *Renilla* luciferase plasmid (phRL-TK) as an internal control for normalization. The luciferase assay was performed with a dual-luciferase reporter assay system (Promega, USA).

### Immunofluorescence staining

To perform immunofluorescence staining for myosin heavy chain, cells were fixed with 4% PFA for 20 min and permeabilized in 0.1% Triton X-100 for 10 min at room temperature. The cells were blocked with the immunostaining blocking buffer (Beyotime, China) for 1 h at room temperature, incubated with primary antibody against myosin heavy chain (1:1,000, Sigma-Aldrich, USA) at 4 °C overnight and then with Goat anti-Mouse IgG (H + L) Highly Cross-Adsorbed Secondary Antibody, Alexa Fluor 594 (1:400, Invitrogen, USA) for 1 h at room temperature. The cells were stained with DAPI for 5 min at room temperature to visualize the nuclei and were finally examined using a fluorescence microscope.

For muscle fibre-type experiments, tibialis anterior (TA) and soleus (Sol) muscle samples were dissected, embedded in optimum cutting temperature (OCT) compound, flash frozen in cold isopentane, and sectioned at 10 μm. Primary antibodies against MHCI, MHCIIa and MHCIIb were obtained from Developmental Studies Hybridoma Bank (DSHB). The following corresponding secondary antibodies were obtained from Thermo-Fisher: Alexa Fluor 594 anti-mouse IgG2b (A-21145), Alexa Fluor 594 anti-mouse IgG1 (A-21125) and Alexa Fluor 594 anti-mouse IgM (A-21044). The TA muscle was stained for MHCIIa and MHCIIb, and the Sol muscle was stained for MHCI.

To count the total number of myofibres, immunofluorescence staining for laminin was performed. The TA muscle was dissected, embedded in OCT compound, flash frozen in cold isopentane, and sectioned at 10 μm. The primary antibody against laminin (L9393) was obtained from Sigma, and the corresponding secondary antibody was Alexa Fluor 488 Goat Anti-Rabbit IgG (H + L) Antibody (A-11008), which was obtained from Invitrogen.

The immunofluorescent staining for MHCI, MHCIIa, MHCIIb and laminin was performed according to a previous report^43^.

### Cas9 vector construction

Cas9 expression vectors were constructed according to a previous study^44^ and were then transfected into C2C12 cells using Nucleofector™ Kits for Mammalian Fibroblasts (Lonza, Germany).

### Southern blot

Genomic DNA was extracted from C2C12 cells or mouse tail tissue and digested with *Hin*dIII (Takara, China) overnight at 37 °C. The digested genomic fragments were separated on a 0.8% agarose gel and transferred to a positively charged nylon membrane (Roche, Germany) for hybridization. The primers for amplifying the digoxigenin-labelled probe were as follows: F: 5′ CCA AAT TCT CTG CCT GGT GG 3′, R: 5′ GCT CCT CTG TGT TCC GTA GA 3′.

The hybridization and detection procedures were performed according to the manufacturer’s instructions (Roche, Germany).

### Protein extraction and Western blot

The total protein was extracted from C2C12 myotubes and gastrocnemius muscle using cell lysis buffer for western blotting and IP (Beyotime, China) according to the manufacturer’s protocol. The quantification of total protein was measured with a BCA Protein Assay Kit (Beyotime, China) according to the manufacturer’s protocol.

The primary antibodies to Pan-Akt, P-Akt (Ser473), P-Akt (Thr308), mTOR, P-mTOR (Ser2448), S6, P-S6 (Ser240/244), P70S6K, P-P70S6K (Thr389), 4E-BP1, 4E-BP1 (Ser65) and GAPDH were all purchased from Cell Signaling Technology. Anti-rabbit IgG HRP (ZSGB-Bio, China) was used as a secondary antibody (1:10,000). The blots were visualized using SuperSignal West Dura Extended Duration Substrate (Thermo scientific, USA).

### ELISA

The amount of total IGF1 in C2C12 myotubes, mouse serum and gastrocnemius muscle were measured using a Mouse/Rat IGF-I Immunoassay kit (R&D systems, USA) according to the manufacturer’s protocol. The concentrations of total IGF1 in C2C12 myobtubes and gastrocnemius muscle were normalized to the total protein respectively.

### Quantitative real-time polymerase chain reaction

Total RNA was extracted from C2C12 myotubes and the gastrocnemius muscles of mice by using TRIzol reagent (Invitrogen, USA). Reverse transcription was performed using 2 μg of RNA. The primers to detect the *Igf1* mRNA expression levels and GAPDH levels are listed below; GAPDH was used as an internal control.

IGF1-F: 5′ GGA CCG AGG GGC TTT TAC TT 3′; IGF1-R: 5′ GTG GGG CAC AGT ACA TCT CC 3′. GAPDH-F: 5′ GTG CCG CCT GGA GAA ACC T 3′; GAPDH-R: 5′ AAG TCG CAG GAG ACA ACC 3′.

The relative expression levels of *Igf1* mRNA in myotubes of single-cell colonies were displayed as fold changes relative to that in WT C2C12 myotubes using the 2^−△△CT^ method. The relative expression levels of *Igf1* mRNA in the gastrocnemius muscles were calculated using the 2^−△CT^ method.

### Haematoxylin and eosin staining (H&E)

The TA and GA were dissected out and fixed in 4% PFA for approximately five days. After dehydration, the samples were embedded in paraffin. Then, 5-μm-thick sections were made and stained with H&E. The CSA of more than 1,500 myofibres from at least five mice per group were measured using image J software.

### Detection of potential off-target sites

Potential off-target sites were screened in the mouse genome using the CRISPR design tool (http://crispr.mit.edu/). The top 10 potential off targets for PCR amplification were shown in Supplementary Table S3, and then the PCR products were examined by Sanger sequencing. The primers for amplifying the potential off targets were shown in Supplementary Table S4.

### Equipment and settings

The images of Fig. 4 were captured using an EVOS FL imaging system (Life technology). The images of Figs 6c, 7c and Supplementary Figure S8 were acquired using a LEICA DM5500 B microscope.

Image processing including brightness and contrast adjustments, cropping, were performed with Adobe Photoshop software. And the brightness and contrast adjustments were applied equally across the entire image and were applied equally to controls.

### Statistical analysis

All values are presented as means ± SEM. The student’s t test was used to determine p values. Statistical significance was defined as p < 0.05.

### Data availability

No datasets were generated or analysed during the current study.

## Electronic supplementary material

Supplementary Information

## References

[1] Palmiter RD (1982). Dramatic growth of mice that develop from eggs microinjected with metallothionein-growth hormone fusion genes. Nature.

[2] Hammer RE (1985). Production of transgenic rabbits, sheep and pigs by microinjection. Nature.

[3] Jaenisch R, Mintz B (1974). Simian virus 40 DNA sequences in DNA of healthy adult mice derived from preimplantation blastocysts injected with viral DNA. Proc. Natl. Acad. Sci. USA.

[4] Jaenisch R (1976). Germ line integration and Mendelian transmission of the exogenous Moloney leukemia virus. Proc. Natl. Acad. Sci. USA.

[5] Gordon JW, Scangos GA, Plotkin DJ, Barbosa JA, Ruddle FH (1980). Genetic transformation of mouse embryos by microinjection of purified DNA. Proc. Natl. Acad. Sci. USA.

[6] Garrick D, Fiering S, Martin DIK, Whitelaw E (1998). Repeat-induced gene silencing in mammals. Nat. Genet..

[7] Wilson C, Bellen HJ, Gehring WJ (1990). Position effects on eukaryotic gene expression. Annu. Rev. Cell Biol..

[8] Brinster RL, Allen JM, Behringer RR, Gelinas RE, Palmiter RD (1988). Introns increase transcriptional efficiency in transgenic mice. Proc. Natl. Acad. Sci. USA.

[9] Palmiter RD, Sandgren EP, Avarbock MR, Allen DD, Brinster RL (1991). Heterologous introns can enhance expression of transgenes in mice. Proc. Natl. Acad. Sci. USA.

[10] Hir HL, Nott A, Moore MJ (2003). How introns influence and enhance eukaryotic gene expression. Trends Biochem. Sci..

[11] King MC, Wilson AC (1975). Evolution at two levels in humans and chimpanzees. Science.

[12] Markljung E (2009). ZBED6, a novel transcription factor derived from a domesticated DNA transposon regulates IGF2 expression and muscle growth. PLoS Biol..

[13] Van Laere AS (2003). A regulatory mutation in IGF2 causes a major QTL effect on muscle growth in the pig. Nature.

[14] Clop A (2006). A mutation creating a potential illegitimate microRNA target site in the myostatin gene affects muscularity in sheep. Nat. Genet..

[15] Banerji J, Rusconi S, Schaffner W (1981). Expression of a β-globin gene is enhanced by remote SV40 DNA sequences. Cell.

[16] Moreau P (1981). The SV40 72 base repair repeat has a striking effect on gene expression both in SV40 and other chimeric recombinants. Nucleic Acids Res..

[17] Bodine SC (2001). Akt/mTOR pathway is a crucial regulator of skeletal muscle hypertrophy and can prevent muscle atrophy *in vivo*. Nat. Cell Biol..

[18] Mourkioti F, Rosenthal N (2005). IGF-1, inflammation and stem cells: interactions during muscle regeneration. Trends Immunol..

[19] Musaro A, Rosenthal N (2002). The role of local insulin-like growth factor-1 isoforms in the pathophysiology of skeletal muscle. Curr. Genomics.

[20] Rommel C (2001). Mediation of IGF-1-induced skeletal myotube hypertrophy by PI(3)K/Akt/mTOR and PI(3)K/Akt/GSK3 pathways. Nat. Cell Biol..

[21] Musaro A (2001). Localized Igf-1 transgene expression sustains hypertrophy and regeneration in senescent skeletal muscle. Nat. Genet..

[22] Hede MS (2012). E-peptides control bioavailability of IGF-1. PloS one.

[23] Shavlakadze T (2010). A growth stimulus is needed for IGF-1 to induce skeletal muscle hypertrophy *in vivo*. J. Cell. Sci..

[24] Shavlakadze T (2006). Rskalpha-actin/hIGF-1 transgenic mice with increased IGF-I in skeletal muscle and blood: impact on regeneration, denervation and muscular dystrophy. Growth Horm. IGF Res..

[25] Coleman ME (1995). Myogenic vector expression of insulin-like growth factor I stimulates muscle cell differentiation and myofiber hypertrophy in transgenic mice. J. Biol. Chem..

[26] Fiorotto ML, Schwartz RJ, Delaughter MC (2003). Persistent IGF-I overexpression in skeletal muscle transiently enhances DNA accretion and growth. FASEB J..

[27] McGrew MJ (1996). Distinct gene expression patterns in skeletal and cardiac muscle are dependent on common regulatory sequences in the MLC1/3 locus. Mol. Cell. Biol..

[28] Donoghue M, Ernst H, Wentworth B, Nadal-Ginard B, Rosenthal N (1988). A muscle-specific enhancer is located at the 3′ end of the myosin light-chain 1/3 gene locus. Genes Dev..

[29] Rosenthal N, Kornhauser JM, Donoghue M, Rosen KM, Merlie JP (1989). Myosin light chain enhancer activates muscle-specific, developmentally regulated gene expression in transgenic mice. Proc. Natl. Acad. Sci. USA.

[30] Wentworth BM, Donoghue M, Engert JC, Berglund EB, Rosenthal N (1991). Paired MyoD-binding sites regulate myosin light chain gene expression. Proc. Natl. Acad. Sci. USA.

[31] Donoviel DB (1996). Analysis of muscle creatine kinase gene regulatory elements in skeletal and cardiac muscles of transgenic mice. Mol. Cell. Biol..

[32] Shield MA, Haugen HS, Clegg CH, Hauschka SD (1996). E-box sites and a proximal regulatory region of the muscle creatine kinase gene differentially regulate expression in diverse skeletal muscles and cardiac muscle of transgenic mice. Mol. Cell. Biol..

[33] Sternberg EA (1988). Identification of upstream and intragenic regulatory elements that confer cell-type-restricted and differentiation-specific expression on the muscle creatine kinase gene. Mol. Cell. Biol..

[34] Johnson JE, Wold BJ, Hauschka SD (1989). Muscle creatine kinase sequence elements regulating skeletal and cardiac muscle expression in transgenic mice. Mol. Cell. Biol..

[35] Siepel A (2005). Evolutionarily conserved elements in vertebrate, insect, worm, and yeast genomes. Genome Res..

[36] Hardison RC, Taylor J (2012). Genomic approaches towards finding cis-regulatory modules in animals. Nat. Rev. Genet..

[37] Temmerman L, Slonimsky E, Rosenthal N (2010). Class 2 IGF-1 isoforms are dispensable for viability, growth and maintenance of IGF-1 serum levels. Growth Horm. IGF Res..

[38] Pais RS (2013). Transcriptome analysis in prenatal IGF1-deficient mice identifies molecular pathways and target genes involved in distal lung differentiation. PloS one.

[39] Ferrando AA (2002). Testosterone administration to older men improves muscle function: molecular and physiological mechanisms. Am. J. Physiol. Endocrinol. Metab..

[40] Urban RJ (1995). Testosterone administration to elderly men increases skeletal muscle strength and protein synthesis. Am. J. Physiol..

[41] Mauras N (1998). Testosterone deficiency in young men: marked alterations in whole body protein kinetics, strength, and adiposity. J. Clin. Endocrinol. Metab..

[42] Wu Y (2007). Identification of androgen response elements in the insulin-like growth factor I upstream promoter. Endocrinology.

[43] Li J (2017). miR-29b contributes to multiple types of muscle atrophy. Nat. Commun..

[44] Yang H, Wang H, Jaenisch R (2014). Generating genetically modified mice using CRISPR/Cas-mediated genome engineering. Nat. Protoc..

