# Supplementary material for: Incorporation of a skeletal muscle-specific enhancer in the regulatory region of Igf1 upregulates IGF1 expression and induces skeletal muscle hypertrophy
Source: Sci Rep. 2018 Feb 9;8:2781. doi: 10.1038/s41598-018-21122-5 (PMC5807547; doi:10.1038/s41598-018-21122-5)

Incorporation of a skeletal muscle-specific enhancer in the regulatory region of *Igf1* upregulates IGF1 expression and induces skeletal muscle hypertrophy

Yunlong Zou[1], Yanjun Dong[2], Qingyong Meng[1], Yaofeng Zhao[1, *], Ning Li[1, *]

1, State Key Laboratory for Agrobiotechnology, China Agricultural University, Beijing 100193, P. R. China

2, College of Veterinary Medicine, China Agricultural University, Beijing 100193, P. R. China

* Correspondence: Ning Li (ninglcau@cau.edu.cn) & Yaofeng Zhao (yaofengzhao@cau.edu.cn) Tel: 0086-10-62734945

Supplementary Table S1: Primers for amplifying the fragments flanking each Cas9 cutting sites.

Primer name	Sequence (5' to 3')	Purpose
igf1(123)-2F	TGTTAGTGGAAGCCTTGGGT	for amplifying fragments flanking the cutting site of Igf1-1, Igf1-2, Igf1-3
igf1(123)-2R	CCCAGATTAGACGCTGCATG	
igf1(678)-2-F	AGTCCAGGCTTCAACATCCA	for amplifying fragments flanking the cutting site of Igf1-4, Igf1-5, Igf1-9
igf1(678)-2-R	TGTTTTCTCACAGTTTAGACCCT	
igf1(45)-2-F	GGTGACAGCCTCATCTCCTA	for amplifying fragments flanking the cutting site of Igf1-6, Igf1-7, Igf1-8
igf1(45)-2-R	GCTTTCCATTGGTCTCTTCCC	

Supplementary Table S2: Primers for detection of site 1, site 2, site 3 series of single-cell colonies and detection of positive GM mice.

Location of primers	Direction	Sequence (5' to 3')
across the 5' junction of site 1 locus	F	ACAGCAGTTCCCCGAGATAG
	R	TTTTCCATTTGATGTTTCTTGA
across the 3' junction of site 1 locus	F	CCGCTCATAACTCAAGTCGC
	R	TCTGACAGGGTTAGCAGACA
across the 5' junction of site 2 locus	F	CATTGGCTGGATTGCTTGGG
	R	TAGGAAAGAGAAGAGCCCGC
across the 3' junction of site 2 locus	F	CCGCTCATAACTCAAGTCGC
	R	CACCAATTCCAAACCTCGGG
across the 5' junction of site 3 locus	F	GCCCTCTTCCTCTGTACCTC
	R	TAGGAAAGAGAAGAGCCCGC
across the 3' junction of site 3 locus	F	GTGCTTTAACTGTCCCCACG
	R	CTGCAGGCCACACATGATC
across the MLC enhancer	F	GGTGACAGCCTCATCTCCTA
	R	GCTTTCCATTGGTCTCTTCCC

Supplementary Table S3: The predicted top 10 potential off-target sites of sgRNA (igf1-9) targeting the site 2 locus.

The mismatches between the sgRNA (igf1-9) targeting the site 2 locus and off-target sites are indicated. The score of each off-target site is proportional to off-target potential.

Off-target sites	Sequence (5' to 3')	Score	Mismatches	Locus
OT-1	AGACACAGTAACATGTCATGCAG	2.6	3MMs [1:2:8]	chr4:+87624996
OT-2	AAAAACAAAAACATGTCATGTGG	1.6	3MMs [1:4:9]	chr9:+37504772
OT-3	TAACACAATTCCATGTCATGGGG	1.4	3MMs [1:10:11]	chr2:-133854410
OT-4	TAGCACAATAATATGTCATGCAG	1.4	3MMs [1:3:12]	chr10:-18410790
OT-5	TAATGCAATTACATGTCATGGAG	1.3	4MMs [1:4:5:10]	chrX:+155655910
OT-6	GGCCACAATTACATGTCATGAGG	1.3	4MMs [1:2:3:10]	chr2:+44403573
OT-7	GCACACAATAACTTGTCATGAAG	1.2	3MMs [1:2:13]	chr7:+114132763
OT-8	CATCACTATACCATGTCATGTGG	1.0	3MMs [3:7:11]	chr9:-73403061
OT-9	GCAAACAAAAACATGTCATGTAG	0.9	4MMs [1:2:4:9]	chr9:-106155022
OT-10	GCACACACAAACATGTCATGGAG	0.9	4MMs [1:2:8:9]	chr12:-14379195

Supplementary Table S4: Primers for amplifying the top 10 potential off-target sites.

Primer name	Sequence (5' to 3')
S2-OT-1-F	TGATGGCACTTGATATGGCC
S2-OT-1-R	CTTTGTAGCAAGCTCGTTTACA
S2-OT-2-F	GGGCAAACTTGAAGAGCAGA
S2-OT-2-R	GGCCAAAGAATAAGAATTACCCC
S2-OT-3-F	AAGATAATCACGGTCGGGCT
S2-OT-3-R	CAAAGGCCATGAGTCTTGCA
S2-OT-4-F	CTGTAGTGTTGGTGCGCATT
S2-OT-4-R	CGCCACCACATTCTCTTGTC
S2-OT-5-F	AAGAACAACCCATCATCTCAGT
S2-OT-5-R	GGAGGCCCACACTTTTAGAC
S2-OT-6-F	TGGGAGACTGTGGCTTGATT
S2-OT-6-R	ACACCCACTTCACACACTCT
S2-OT-7-F	ATTCTGCACGTGGTCATCTG
S2-OT-7-R	GGCCTCAGAGCACCTAATGT
S2-OT-8-F	CGCTATATCATGACTCCCTGC
S2-OT-8-R	CGGGAGTTTGTTACATGGACC
S2-OT-9-F	TCTCTGGTGGGTCTCCTACA
S2-OT-9-R	ACTTCCTCTCCTGTGTCTGC
S2-OT-10-F	TCCTTATCTTTCCTTCCTTCCGA
S2-OT-10-R	CCCTAAGGCCAAGTCTCACA

Supplementary Figure S1: Confirmation of successful homologous recombination in a pool of C2C12 cells sorted by flow cytometry.

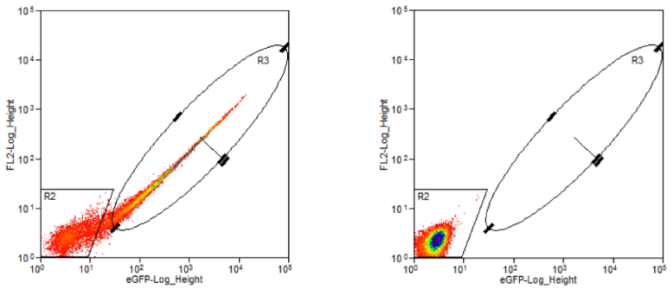

The GFP-expressing vector (EGFPN1), the donor vector and plasmid expressing both the Cas9 and sgRNAs targeting each of the three candidate loci were co-transfected into C2C12 cells, which were harvested two days after transfection for flow cytometry to select GFP$^+$ cells. The sorted cell populations are indicated by a black oval frame (left). Flow cytometry analysis of the WT C2C12 cells without any manipulation (right).

Supplementary Figure S2: Modification of the PX330 vector for enrichment of targeted cells by flow cytometry.

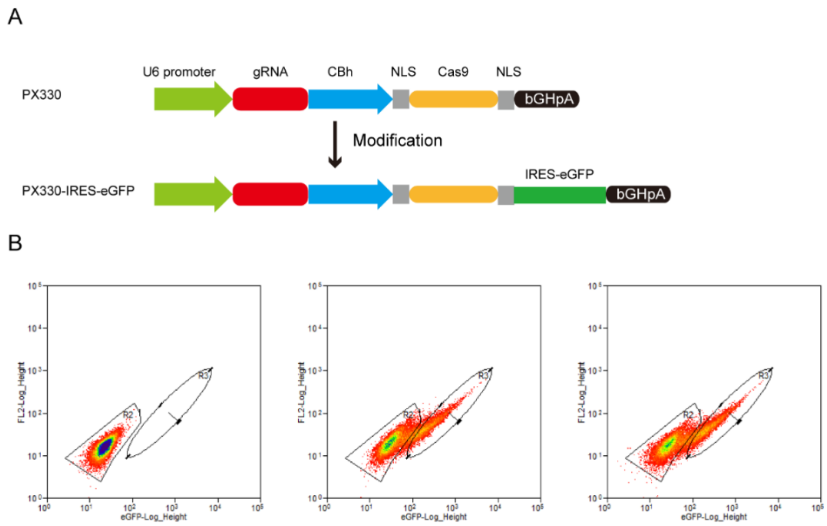

(A) Modification of the PX330 plasmid by linking a green fluorescent protein (GFP) expressing element to the Cas9 protein-coding cassette via an IRES sequence. The new vector was named PX330-IRES-eGFP.

(B) Enrichment of the GFP$^+$ cell populations by flow cytometry. (Left, WT C2C12 cells without any manipulation; middle, targeting the site 2 locus; right, targeting the site 3 locus.) The sorted cell populations are indicated by a black oval frame.

Supplementary Figure S3: Detection of the off-target effects of sgRNA targeting the site 2 locus in C2C12 single-cell colonies.

```
OT-1:
WT:   CTTGGGCTCTAAAGTTGGGAGGGATGAAGAAGACACAGTAACATGTCATGCAGCTCTGCCCTCAATAGAACACACACACCATG
2-13: CTTGGGCTCTAAAGTTGGGAGGGATGAAGAAGACACAGTAACATGTCATGCAGCTCTGCCCTCAATAGAACACACACACCATG
2-14: CTTGGGCTCTAAAGTTGGGAGGGATGAAGAAGACACAGTAACATGTCATGCAGCTCTGCCCTCAATAGAACACACACACCATG
2-30: CTTGGGCTCTAAAGTTGGGAGGGATGAAGAAGACACAGTAACATGTCATGCAGCTCTGCCCTCAATAGAACACACACACCATG
2-57: CTTGGGCTCTAAAGTTGGGAGGGATGAAGAAGACACAGTAACATGTCATGCAGCTCTGCCCTCAATAGAACACACACACCATG

OT-2
WT:   TGGCATTGTTTTTTAAGGGAAAAAAAAAACAAAAACAAAAACATGTCATGTGGGTTGTCTAAAAATAAAGTGCATTTAAATCC
2-13: TGGCATTGTTTTTTAAGGGAAAAAAAAAACAAAAACAAAAACATGTCATGTGGGTTGTCTAAAAATAAAGTGCATTTAAATCC
2-14: TGGCATTGTTTTTTAAGGGAAAAAAAAAACAAAAACAAAAACATGTCATGTGGGTTGTCTAAAAATAAAGTGCATTTAAATCC
2-30: TGGCATTGTTTTTTAAGGGAAAAAAAAAACAAAAACAAAAACATGTCATGTGGGTTGTCTAAAAATAAAGTGCATTTAAATCC
2-57: TGGCATTGTTTTTTAAGGGAAAAAAAAAACAAAAACAAAAACATGTCATGTGGGTTGTCTAAAAATAAAGTGCATTTAAATCC

OT-3
WT:   CAGGTGATACTGTGAGTCACTAGCATGGGTTAACACAATTCCATGTCATGGGGGCCAAGAAAGGAGCTAGGAGATAAAAGAAT
2-13: CAGGTGATACTGTGAGTCACTAGCATGGGTTAACACAATTCCATGTCATGGGGGCCAAGAAAGGAGCTAGGAGATAAAAGAAT
2-14: CAGGTGATACTGTGAGTCACTAGCATGGGTTAACACAATTCCATGTCATGGGGGCCAAGAAAGGAGCTAGGAGATAAAAGAAT
2-30: CAGGTGATACTGTGAGTCACTAGCATGGGTTAACACAATTCCATGTCATGGGGGCCAAGAAAGGAGCTAGGAGATAAAAGAAT
2-57: CAGGTGATACTGTGAGTCACTAGCATGGGTTAACACAATTCCATGTCATGGGGGCCAAGAAAGGAGCTAGGAGATAAAAGAAT

OT-4
WT:   CAGAAAGGTGTCATGGACAACCTCTGACAATAGCACAATAATATGTCATGCAGTTTTTATAAGCATTGGGTTTTTCCAATGGC
2-13: CAGAAAGGTGTCATGGACAACCTCTGACAATAGCACAATAATATGTCATGCAGTTTTTATAAGCATTGGGTTTTTCCAATGGC
2-14: CAGAAAGGTGTCATGGACAACCTCTGACAATAGCACAATAATATGTCATGCAGTTTTTATAAGCATTGGGTTTTTCCAATGGC
2-30: CAGAAAGGTGTCATGGACAACCTCTGACAATAGCACAATAATATGTCATGCAGTTTTTATAAGCATTGGGTTTTTCCAATGGC
2-57: CAGAAAGGTGTCATGGACAACCTCTGACAATAGCACAATAATATGTCATGCAGTTTTTATAAGCATTGGGTTTTTCCAATGGC

OT-5
WT:   GAATCGTTTTTGTCGGGCAGAGGCCTGAATTAATGCAATTACATGTCATGGAGCTTTCTAATAATGGAGGATGGTGAGCTTTA
2-13: GAATCGTTTTTGTCGGGCAGAGGCCTGAATTAATGCAATTACATGTCATGGAGCTTTCTAATAATGGAGGATGGTGAGCTTTA
2-14: GAATCGTTTTTGTCGGGCAGAGGCCTGAATTAATGCAATTACATGTCATGGAGCTTTCTAATAATGGAGGATGGTGAGCTTTA
2-30: GAATCGTTTTTGTCGGGCAGAGGCCTGAATTAATGCAATTACATGTCATGGAGCTTTCTAATAATGGAGGATGGTGAGCTTTA
2-57: GAATCGTTTTTGTCGGGCAGAGGCCTGAATTAATGCAATTACATGTCATGGAGCTTTCTAATAATGGAGGATGGTGAGCTTTA

OT-6
WT:   GCAGGAGACATTGCAGAGGCCAGTAGGAAAGGCCACAATTACATGTCATGAGGAATTGGCAATAGTTCAACCTCATGAAGAC
2-13: GCAGGAGACATTGCAGAGGCCAGTAGGAAAGGCCACAATTACATGTCATGAGGAATTGGCAATAGTTCAACCTCATGAAGAC
2-14: GCAGGAGACATTGCAGAGGCCAGTAGGAAAGGCCACAATTACATGTCATGAGGAATTGGCAATAGTTCAACCTCATGAAGAC
2-30: GCAGGAGACATTGCAGAGGCCAGTAGGAAAGGCCACAATTACATGTCATGAGGAATTGGCAATAGTTCAACCTCATGAAGAC
2-57: GCAGGAGACATTGCAGAGGCCAGTAGGAAAGGCCACAATTACATGTCATGAGGAATTGGCAATAGTTCAACCTCATGAAGAC

OT-7
WT:   AGCCAGCCACATAAGTGAAAGCCATCAACTGCACACAATAACTTGTCATGAAGACTCGATATTGGAGAGGGTAGCAGTTGGCC
2-14: AGCCAGCCACATAAGTGAAAGCCATCAACTGCACACAATAACTTGTCATGAAGACTCGATATTGGAGAGGGTAGCAGTTGGCC
2-30: AGCCAGCCACATAAGTGAAAGCCATCAACTGCACACAATAACTTGTCATGAAGACTCGATATTGGAGAGGGTAGCAGTTGGCC
2-57: AGCCAGCCACATAAGTGAAAGCCATCAACTGCACACAATAACTTGTCATGAAGACTCGATATTGGAGAGGGTAGCAGTTGGCC

OT-8
WT:   TTTGGGACGAAAGTTAACCATTTACCCCACCATCACTATACCATGTCATGTGGCACCCTCGTGTACCACAATAAAAGCATCAT
2-13: TTTGGGACGAAAGTTAACCATTTACCCCACCATCACTATACCATGTCATGTGGCACCCTCGTGTACCACAATAAAAGCATCAT
2-14: TTTGGGACGAAAGTTAACCATTTACCCCACCATCACTATACCATGTCATGTGGCACCCTCGTGTACCACAATAAAAGCATCAT
2-30: TTTGGGACGAAAGTTAACCATTTACCCCACCATCACTATACCATGTCATGTGGCACCCTCGTGTACCACAATAAAAGCATCAT
2-57: TTTGGGACGAAAGTTAACCATTTACCCCACCATCACTATACCATGTCATGTGGCACCCTCGTGTACCACAATAAAAGCATCAT

OT-9
WT:   AGGTAGCCTGGCATTCCTTTTTAAGCAAAAGCAAACAAAAACATGTCATGTAGGTTCTTGAAAAATAAACTGAAAAACAAACA
2-13: AGGTAGCCTGGCATTCCTTTTTAAGCAAAAGCAAACAAAAACATGTCATGTAGGTTCTTGAAAAATAAACTGAAAAACAAACA
2-14: AGGTAGCCTGGCATTCCTTTTTAAGCAAAAGCAAACAAAAACATGTCATGTAGGTTCTTGAAAAATAAACTGAAAAACAAACA
2-30: AGGTAGCCTGGCATTCCTTTTTAAGCAAAAGCAAACAAAAACATGTCATGTAGGTTCTTGAAAAATAAACTGAAAAACAAACA
2-57: AGGTAGCCTGGCATTCCTTTTTAAGCAAAAGCAAACAAAAACATGTCATGTAGGTTCTTGAAAAATAAACTGAAAAACAAACA

OT-10
WT:   GTTATTACTAATGTGAATGTGTTATTATATGCACACACAAACATGTCATGGAGAAGCATAAAAATTATAACTCTACATGATAT
2-13: GTTATTACTAATGTGAATGTGTTATTATATGCACACACAAACATGTCATGGAGAAGCATAAAAATTATAACTCTACATGATAT
2-14: GTTATTACTAATGTGAATGTGTTATTATATGCACACACAAACATGTCATGGAGAAGCATAAAAATTATAACTCTACATGATAT
2-30: GTTATTACTAATGTGAATGTGTTATTATATGCACACACAAACATGTCATGGAGAAGCATAAAAATTATAACTCTACATGATAT
2-57: GTTATTACTAATGTGAATGTGTTATTATATGCACACACAAACATGTCATGGAGAAGCATAAAAATTATAACTCTACATGATAT
```

Sanger sequencing of the top 10 potential off-target sites in four randomly selected site 2 series of single-cell colonies.

Supplementary Figure S4: Detection of the Akt phosphorylation levels in the gastrocnemius muscle of two-month old female mice.

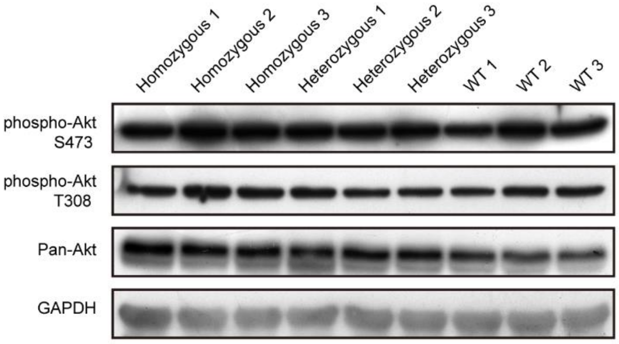

Western blot analysis of the Akt phosphorylation levels in the gastrocnemius muscle of two-month old female GM mice and WT littermates. Antibodies against phosphorylated Akt (Ser473), Akt (Thr308), and total Akt were used. GAPDH was used as a loading control. All gels/blots were run under the same experimental conditions. Shown are cropped gels/blots (Full-length gels/blots with indicated cropping lines are shown in Supplementary Figure S10).

Supplementary Figure S5: Differentiation index analysis of the site 2 single-cells colony series and WT C2C12 cells.

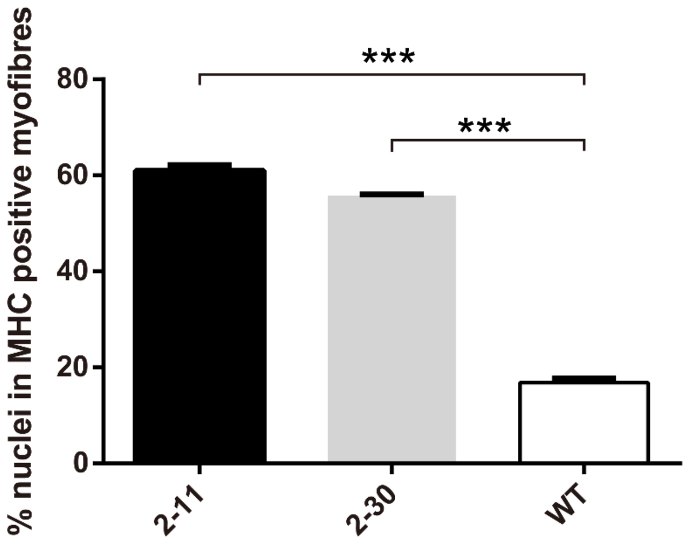

The differentiation index was calculated as the percentage of nuclei in MHC-positive cells by dividing the number of nuclei in MHC-positive cells by the total number of nuclei. Data were obtained from three independent experiments. Bars depict mean values, and error bars represent the SEM. ***P < 0.00001.

Supplementary Figure S6: Detection of IGF1/Akt pathway activity in the gastrocnemius muscle of one-month-old mice.

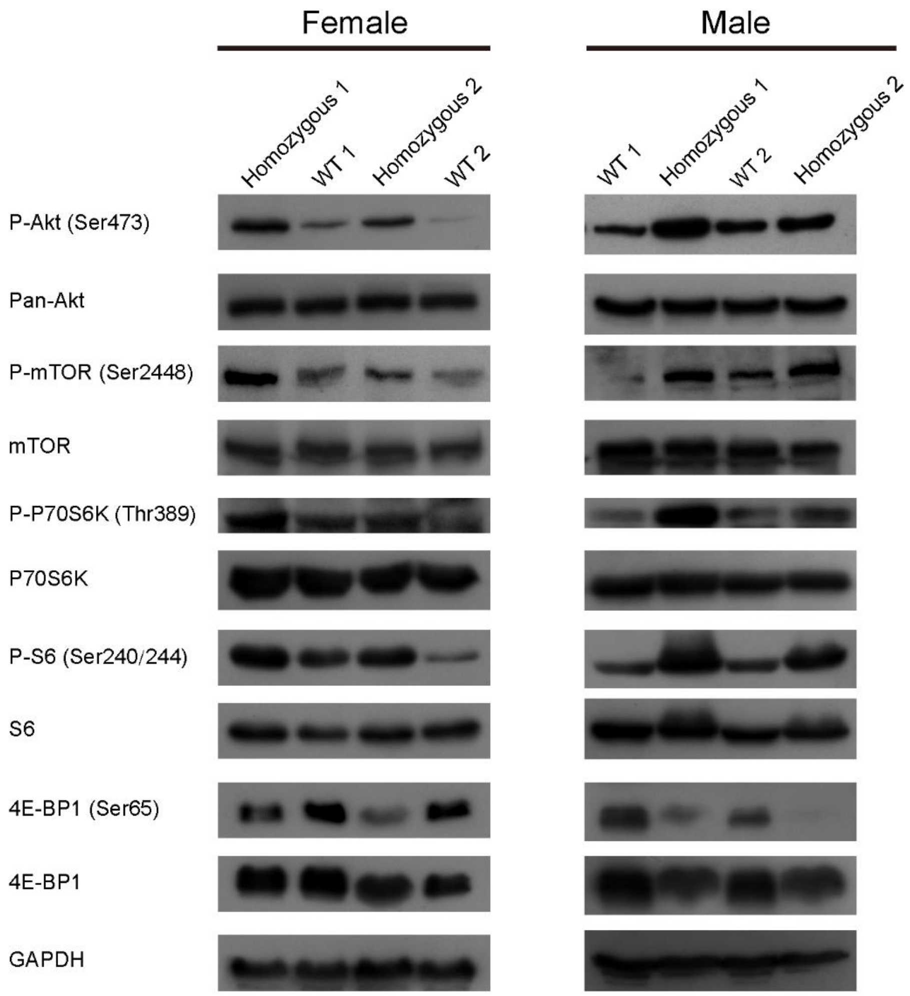

Western blot analysis showed that the IGF1/Akt pathway was stimulated in the gastrocnemius muscle of one-month-old male and female GM mice compared with age- and sex-matched WT littermates. All gels/blots were run under the same experimental conditions. Shown are cropped gels/blots (Full-length gels/blots with indicated cropping lines are shown in Supplementary Figure S10).

Supplementary Figure S7: Analysis of the total number of myofibres in the tibialis anterior (TA) muscle.

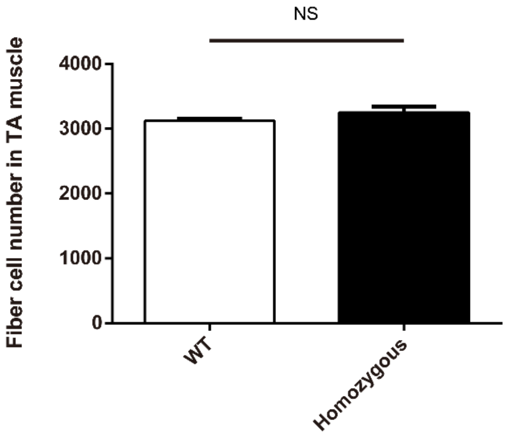

Comparison of the total myofibre number in the TA muscle of two-month-old GM mice and sex-matched WT littermates (n = 5 for the homozygous GM mice and n = 4 for the WT littermates). NS, not significant.

Supplementary Figure S8: Upregulated IGF1 preferentially exerts a hypertrophic effect on MHC type IIb fibres.

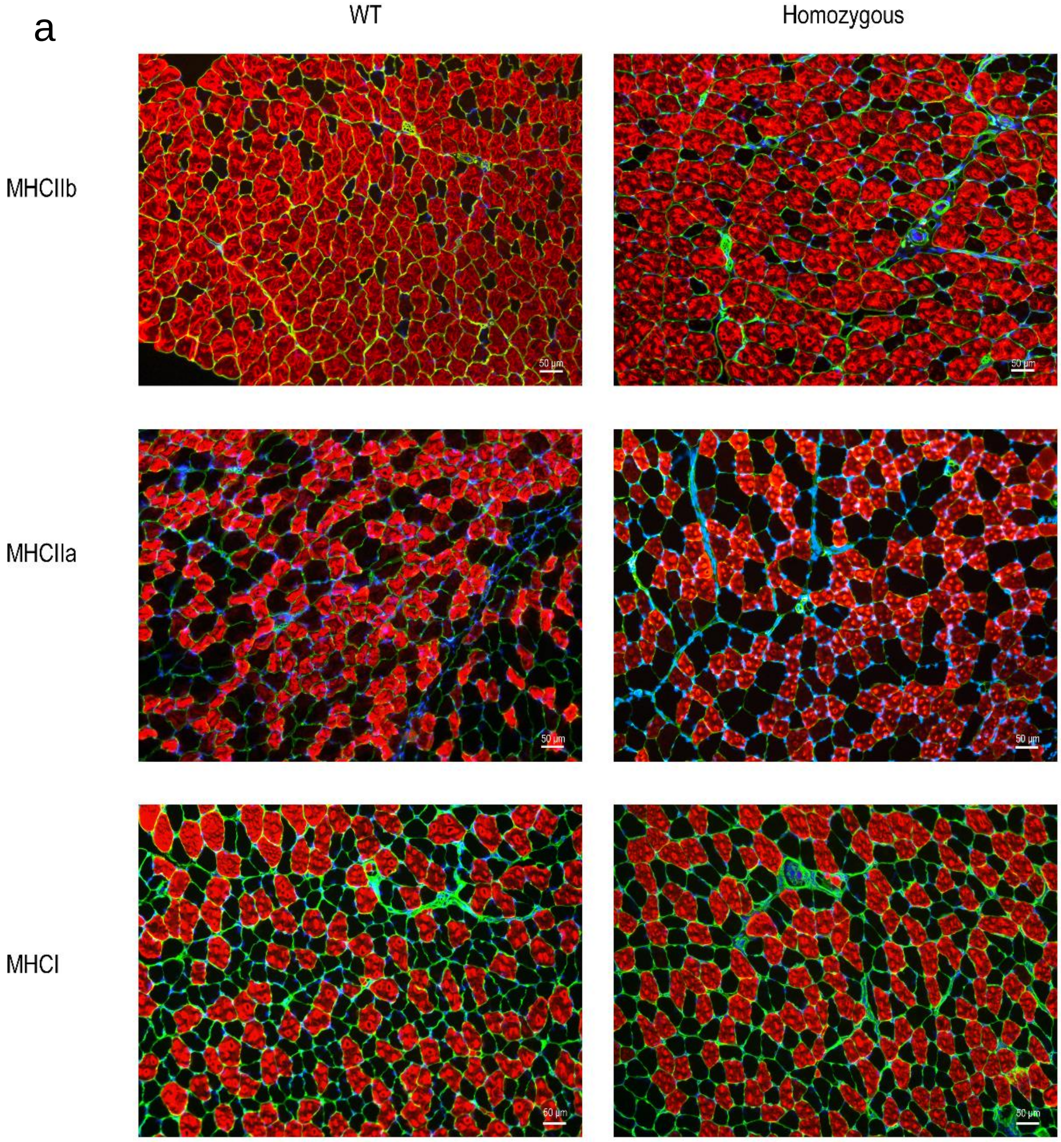

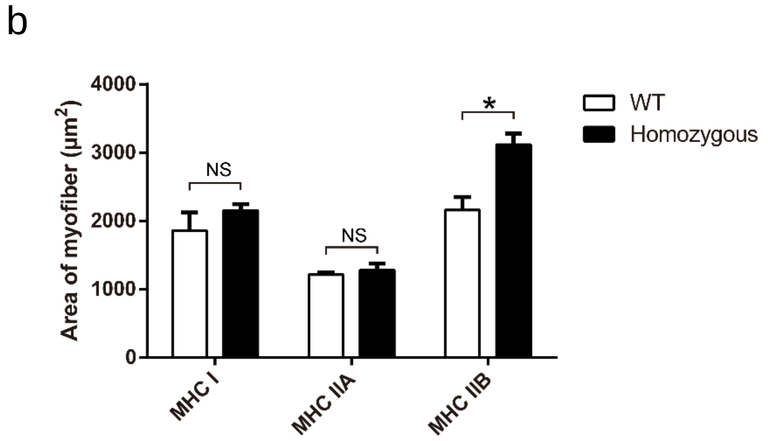

(A) Representative immunofluorescence images of MHCI, MHCIIa, and MHCIIb staining. Cross-sections of the TA muscle were stained with antibodies against MHCIIa and MHCIIb, and cross-sections of the Sol muscle were stained with an antibody against MHCI. Red represents specific types of myofibres detected. Green represents laminin, and blue represents DAPI. Scale bar: 50 µm.
(B) CSA analysis of myofibres of MHC type I, IIa and IIb (n = 3 per group).
Bars depict mean values, and error bars represent the SEM. *P < 0.05; NS, not significant.

Supplementary Figure S9: Detection of S6 phosphorylation levels (Ser240/244) in the EDL and Sol muscles of one-month-old mice.

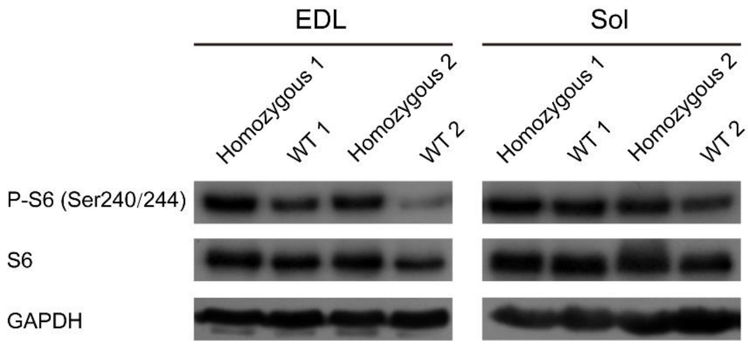

Western blot analysis showed that the S6 phosphorylation levels (Ser240/244) were upregulated in the EDL muscles of GM mice, whereas no significant differences in the S6 phosphorylation levels (Ser240/244) were detected in the Sol muscles of GM mice compared with WT littermates. All gels/blots were run under the same experimental conditions. Shown are cropped gels/blots (Full-length gels/blots with indicated cropping lines are shown in Supplementary Figure S10).

Supplementary Figure S10: Uncropped scans of Western blots

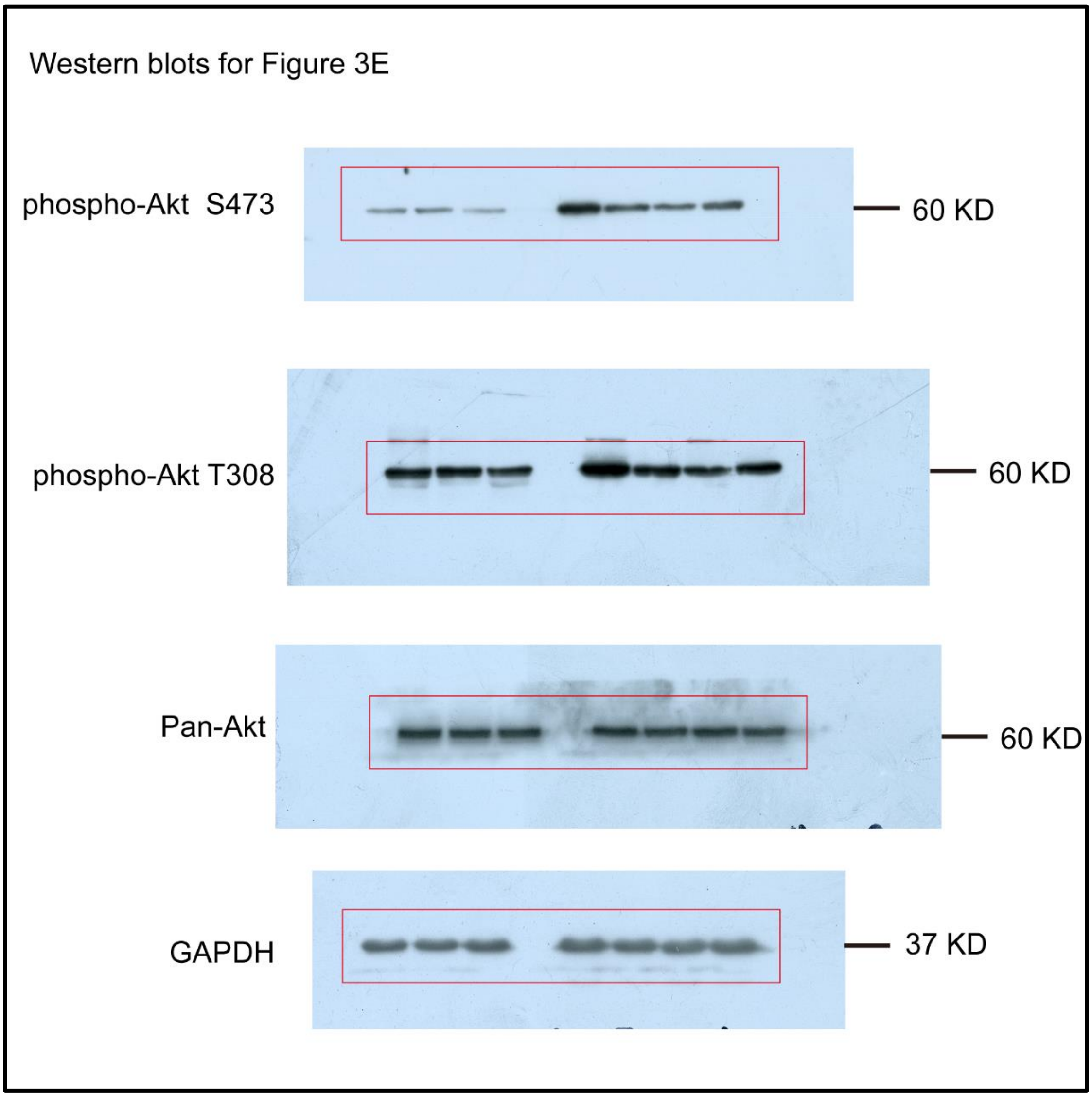

Western blots for Figure 5D

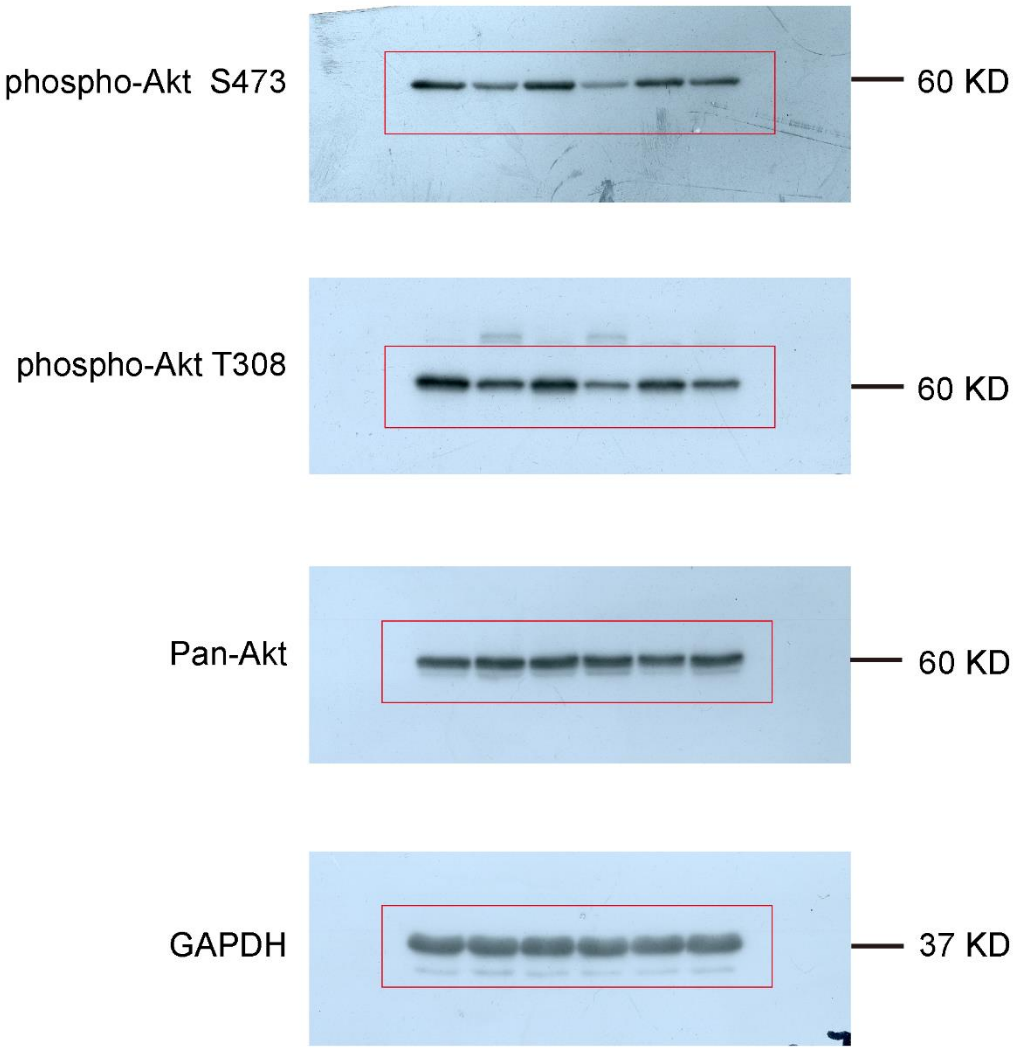

Western blots for Supplementary Figure S4

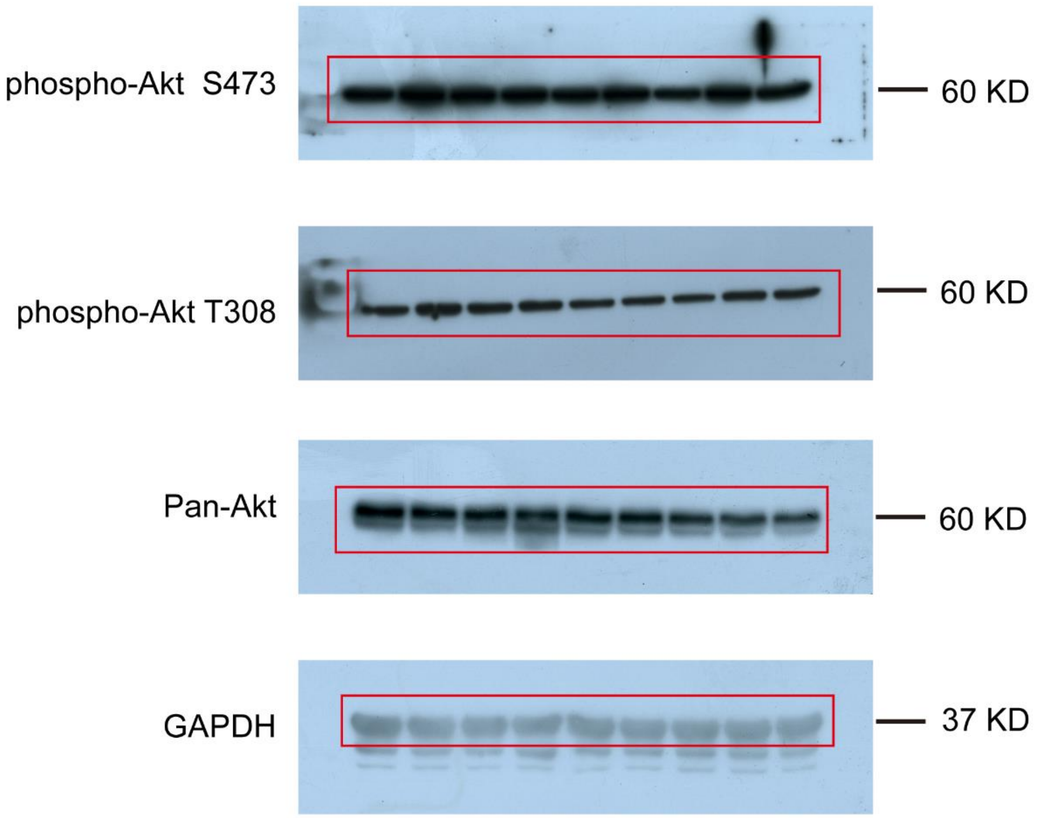

Western blots for male mice in Supplementary Figure S6

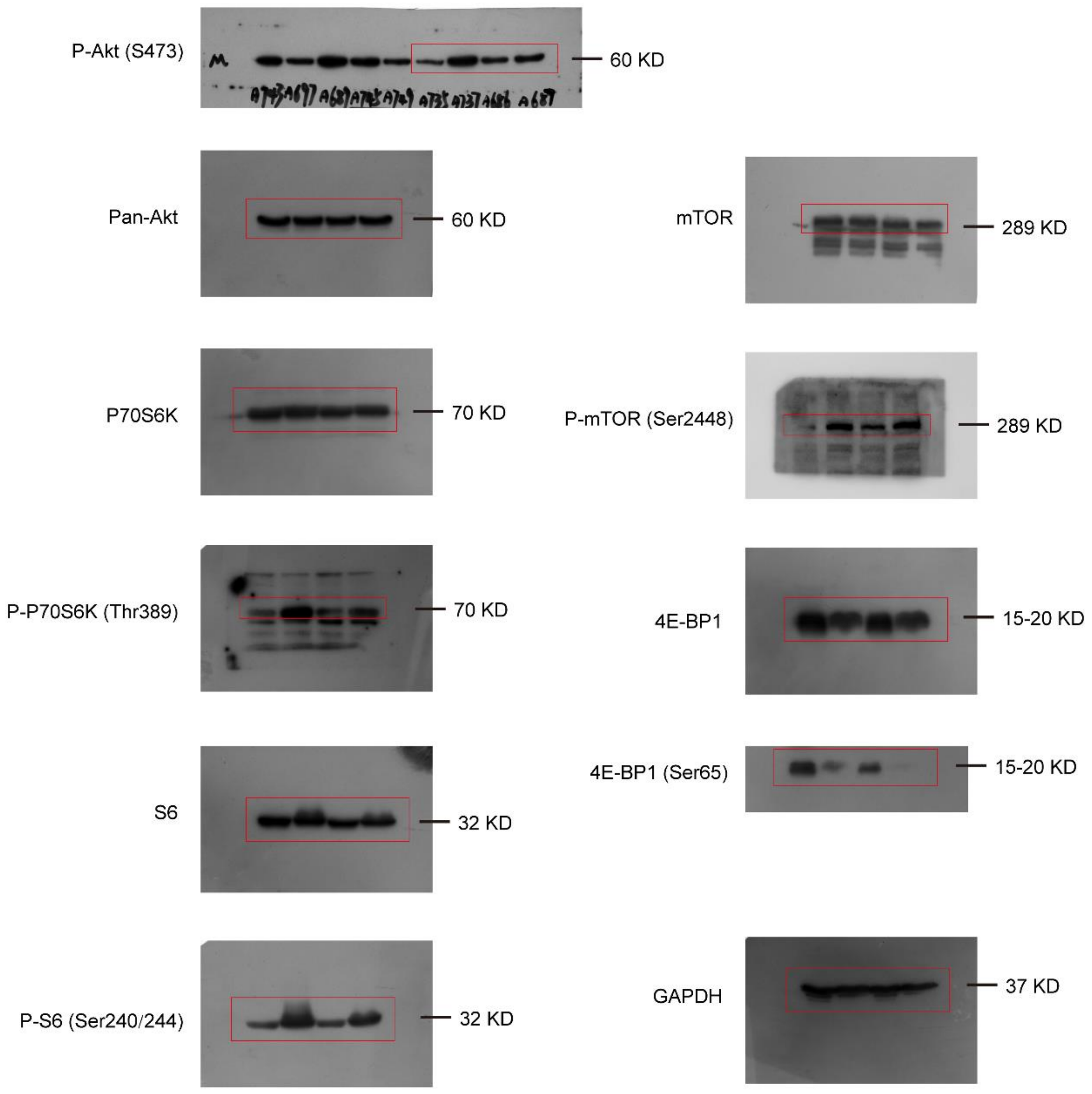

Western blots for female mice in Supplementary Figure S6

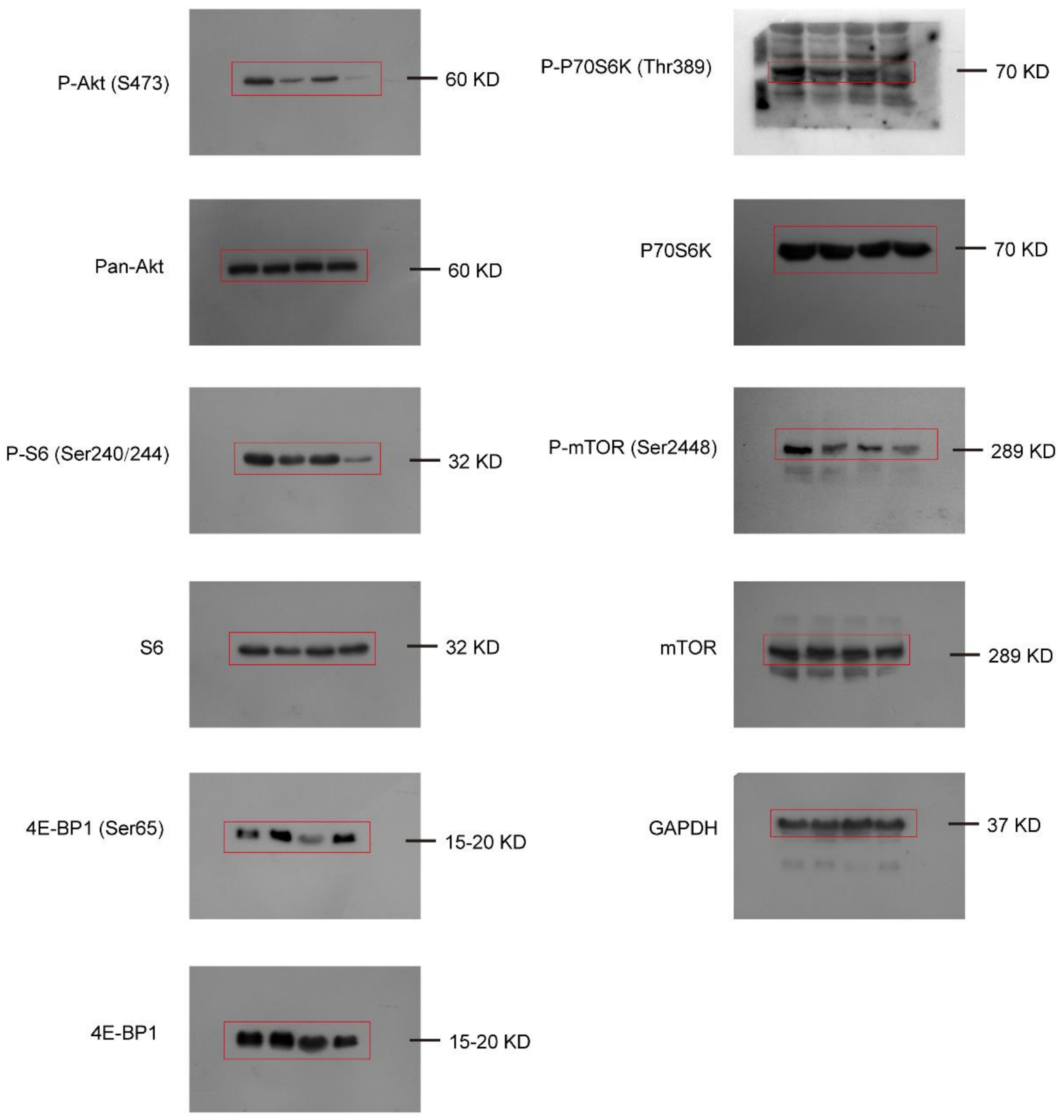

Western blots for Supplementary Figure S9

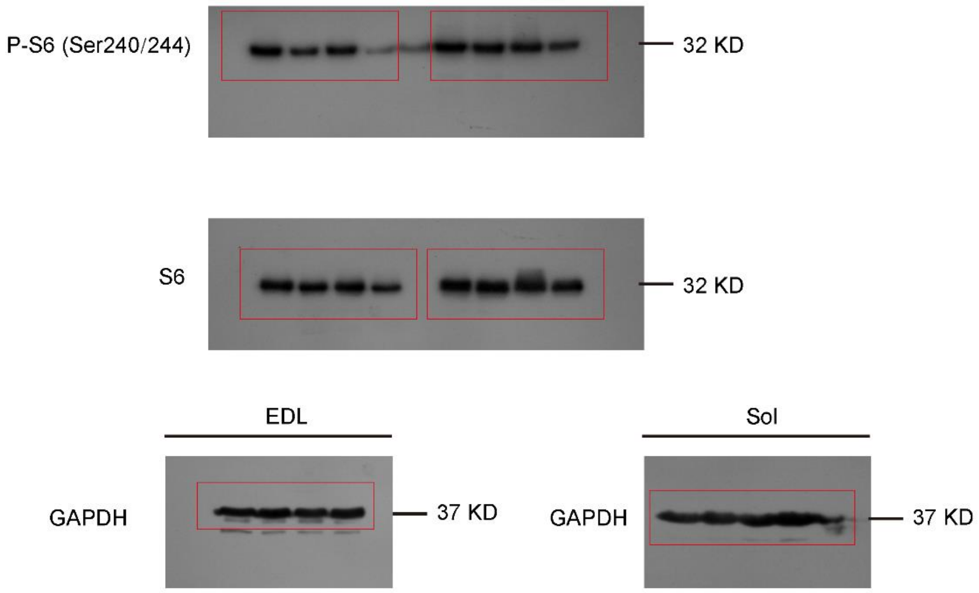

Supplement: Supplementary file 1 — Supplementary Information [file 41598_2018_21122_MOESM1_ESM.pdf]
